# Supplementary material for: Comparing membrane sweep and cervical massage in preventing the need for formal labour induction in uncomplicated pregnancy at term: Secondary analysis of a randomized controlled trial
Source: PLoS One. 2025 May 20;20(5):e0324172. doi: 10.1371/journal.pone.0324172 (PMC12091732; doi:10.1371/journal.pone.0324172)
Supplement: S4 Data — (XLSX) [file pone.0324172.s004.docx]

Madugalle TMSSB

cinla007@yahoo.com

Research Proposal Summary

**Introduction**: Adjuncts for induction (membrane sweep or cervical massage) can be used to prevent formal induction with all its inherent risks and to reduce induction to delivery time. There are no studies comparing the efficacy and acceptability of membrane sweep vs cervical massage. **Methods**: 300 women with uncomplicated antenatal period without any indication for early delivery will be randomly allocated to undergo membrane sweep or cervical massage or neither as the control group. Membrane sweep or cervical massage will be performed at 39^th^, week and in 1 week if spontaneous labour doesn’t follow. All will be followed up till discharge after delivery. Number going into spontaneous labour, number needing formal induction, intervention to delivery time, vaginal delivery rate by 40 and 40+6 weeks of gestation, cesarean section rate, oxytocin augmentation rate, hyperstimulation, post-partum bleeding, maternal fever, hospital stay, APGAR at 5 minutes and maternal acceptability will be assessed. Comparisons between two intervention groups, between each intervention group and control group will be performed. **Results:** Comparative effectiveness and acceptability of membrane sweep and cervical massage will be determined based on a null-hypothesis of non-significant difference.

**Comparing Effectiveness of and maternal Acceptability with uSing cErvicaL massage vs mEmbrane Sweep for cervical ripening in pregnant women at 38th week of geStation at a tertiary care unit (CEASELESS):
A randomized controlled clinical trial**

# Section list

1. **Section 1**
   1. General details
2. **Section 2**
   1. Introduction
   2. Literature review
   3. Objectives and aims
   4. Methods
   5. Financial arrangements
   6. Ethical considerations
   7. Work plan and timeline
   8. Annexures
      1. Consent form Sinhala version
      2. Consent form Tamil version
      3. Data sheet
   9. Reference
   10. Affirmation
3. **Section 3**
   1. Supervisor’s recommendation
4. **Section 4**
   1. Submission

# Section 1

# General details

1. Name of trainee

Dr. Thennakoon Mudiyanselage Salila Sameera Bandara Madugalle

1. Name of supervisor

Dr. Chandana Jayasundara

Consultant Obstetrician and Gynecologist

Professorial Unit

De Soysa Hospital for Women

Colombo 8

1. Training Centre

De Soysa Hospital for Women - Year 1

Colombo North Teaching Hospital – Year 2

# section 2

# CHAPTER 1 - Introduction

Labour is defined as the presence of painful, regular, intermittent contractions of increasing intensity, frequency and duration resulting in progressive cervical dilatation and effacement. Labour consists of three stages. The first stage of labour denotes the period of cervical dilatation from 0cm to 10cm. The first stage of labour can be subdivided into a latent phase and an active phase. The latent phase is the period of cervical dilatation from 0cm to 4cm. The active phase is from a cervical dilatation of 4cm to 10cm. The second stage of labour is from full dilatation to the delivery of the baby. The third stage is from the delivery of the baby to the delivery of the placenta and membranes ^1^.

Assessment of labour includes assessment of the baby, mother and the process of labour. Assessment of the labour process includes assessment of descent of presenting fetal part vaginally and abdominally, frequency and strength of uterine contractions and cervical status. This requires a standardized approach to effectively manage labour and for better communication between team members. Modified Bishop’s Score is such a tool which is highly predictive of the likelihood of vaginal delivery and correlates cervical status during latent phase of labour well to the likelihood of a vaginal delivery. A score ≥7 denotes a ripe cervix favorable for and predictive of a successful vaginal delivery in the presence of adequate contractions ^2^.

Induction of labour is the artificial stimulation of the uterus to start labour and consists of simultaneous initiation of uterine contractions and cervical ripening ^3^. Cervical ripening involves complex interactions of prostaglandin with cervical cells and matrix, leading to dilation, shortening and softening of the cervix, which in turn increases the likelihood of vaginal delivery ^4^.

Sometimes, the phrase ‘induction of labour’ is used to denote only the initiation of contractions. And the phrase ‘cervical ripening’ is used to denote a separate process of achieving a Modified Bishop’s Score of ≥7. These definitions lead to confusion when confronted with accepted definitions of labour and the fact that methods of induction are all administered to a non-contractile uterus and a cervix with a Modified Bishop’s Score ≤6; A.K.A an un-ripe cervix to achieve a score of ≥7.

Induction of labour is a relatively common procedure, and its frequency varies from institution to institution. According to WHO Global Survey on maternal and perinatal health, 9.6% of all deliveries start with an induction worldwide, and Sri Lanka tops the list at 35.5% ^5^.

Methods of induction of labour are classified into mechanical and pharmacological categories. Mechanical methods of induction are membrane sweeping, cervical massage and balloon catheter. The principle of these mechanical methods of induction is to potentiate a local release of endogenous prostaglandin by physically stimulating the cervix. Balloon catheter, while not recommended for routine use by NICE, is widely utilized in Sri Lanka ^6^. A commonly used balloon catheter induction protocol is to insert 18F Foley catheter through the cervical canal so that the inflatable balloon lies beyond the internal cervical OS. Then the balloon is inflated with 30-60cc of water. The catheter tube is then anchored to the patient’s thigh with gentle traction. Balloon catheter is left inside the cervical canal up to a maximum of 48 hours ^4^. Whenever the catheter falls or at the end of the 48 hours an assessment of Modified Bishops’ Score is conducted. In case of failure to induce labour at 48 hours, individualized assessment and a different method of induction or caesarian section is considered. Membrane sweeping and cervical massage are considered as adjuncts for induction and not as primary induction methods. Membrane sweeping involves insertion of examiner’s finger past the internal cervical os and separation of the chorio-amnion from the lower uterine segment with three circumferential movements ^7^. A minimum cervical dilatation adequate to insert one finger is a prerequisite. Recommendation is to offer membrane sweep at 39^th^ and 40^th^ weeks of gestation to nulliparous women and at 40^th^ week of gestation to multiparous women^6^. Cervical massage is mechanical stimulation of surface of cervix with the examiner’s middle finger and index finger for 15-30 seconds ^4^. This does not require the cervix to be minimally dilated. Cervical massage is an alternative that can be offered instead of membrane sweep with a similar regimen. Pharmacological methods of induction introduce exogenous prostaglandin achieving high local concentrations at the cervix. Vaginal prostaglandin is a commonly used pharmacological method of induction. Sri Lankan guidelines recommend one cycle of 3mg prostaglandin tablet or 0.5mg vaginal gel to be inserted (two doses 6 hours apart) ^8^. The second cycle is administered 24 hours after the end of the first cycle if indicated following assessment on an individual basis. Prostaglandin interacts with the cells and matrix of cervix changing its constitution, ripening the cervix. In addition, prostaglandin acts on cellular receptors of myometrial cells triggering rhythmic uterine contractions. Uterine contractions spread from uterine fundus to the lower uterine segment, pressing the presenting fetal part against the cervix, stretching it ^9^. Cervical stretching causes pulsatile release of oxytocin from the posterior pituitary (Ferguson reflex) ^10^. Oxytocin augments rhythmic uterine contractions and potentiates prostaglandin action on myometrium. These events start a cascade of reactions that culminates in induction of labour and subsequent vaginal delivery.

Induction of labour is a clinical decision made when the risks of waiting for spontaneous onset of labour outweighs the risks of shortening the duration of pregnancy. While the aim of induction is to achieve a vaginal birth as naturally as possible, it is in essence an interference in the natural process of pregnancy and labour, making it potentially less efficient, more painful and more problematic compared to spontaneous onset of labour ^6^. Once the decision to induce is taken, the method of induction needs to be agreed upon. There is ample evidence on methods of induction of labour and their inherent risks and complications. Pharmacological induction with vaginal prostaglandin carries a 2.4% risk of uterine rupture compared to that of 0.77% in mechanical methods following one past caesarian section ^11^. Mechanical methods have a lower risk of uterine hyperstimulation and no significant difference in rate of intra partum infections ^12, 13, 14^.

Extensive evidence is available on safety, feasibility, effectiveness, acceptability and maternal satisfaction of mechanical and pharmacological methods of induction. There are situations where use of formal pharmacological induction is cautioned, such as fetal growth restriction and past uterine injury. In such instances, mechanical induction is the only way to go. Induction of labour is more painful, less efficient and more problematic compared to spontaneous onset ^1^. Therefore, adjuncts for induction (membrane sweep or cervical massage) can be used to prevent formal induction with all its inherent risks (Number needed to treat – 8) and to reduce induction to delivery time ^7^. However, if labour is induced, adequate pain relief, morning-time induction, good supportive attitudes of antenatal and labour ward staff and provision of adequate information exponentially improves maternal satisfaction and acceptability of induction of labour ^1, 3, 7, 5, 15^.

The theoretical framework of acceptability of clinical intervention in seven-fold. It consists of, affective attitude, burden, perceived effectiveness, ethicality, intervention coherence, opportunity costs, and self-efficacy. Affective attitude means the emotional reaction to the intervention, for example “I feel scared when I think about the intervention”. Burden refers to the effort or toll taken by the health care system for the implementation of an intervention. The effectiveness of an intervention can be determined through clinical trials. But perceived effectiveness refers to the impression the mothers have about the effectiveness of the intervention. Ethicality can be upheld by strict adherence of research ethical principles. Coherence of an intervention is the quality of being logical and consistent. Opportunity cost means the benefits lost elsewhere due to implementation of one intervention. Self-efficacy of an intervention refers to the ability of an intervention to achieve its aims on its own without external compounding factors.

All these aspects must be taken into consideration regarding assessing he acceptability of an intervention. Affective attitude and perceived effectiveness can be inquired from the subjects. Burden, ethicality, intervention coherence, opportunity cost and self-efficacy can be measured with carefully designed clinical research^33^.

This randomized controlled study aims to compare the effectiveness and maternal acceptability of membrane sweep vs cervical massage in ripening the uterine cervix, establishing spontaneous labour, in extension, preventing formal induction of labour.

# CHAPTER 2 – LITERATURE REVIEW

There are only a few studies on effectiveness, risks, benefits of membrane sweeping to ripen the cervix, while research on cervical massage as a method of cervical ripening where sweep is not possible or as an alternative to membrane sweeping is practically nonexistent. Comparative evidence to support an informed decision between membrane sweep and cervical massage was not available. Available non-comparative research on safety, efficacy, risk and benefits of individual mechanical methods of induction are highly conflicting.

Cervical ripening needs prostaglandin to give rise to specific changes in cervical tissue. In 1977, amniotomy, vaginal examination with or without membrane sweep was compared with regards to peripheral plasma prostaglandin levels before and five minutes after amniotomy, vaginal examination with sweeping of the fetal membranes and vaginal examination without sweeping of the membranes. Each arm showed a significant rise in plasma prostaglandin levels, while the subjects who had the membrane sweep showed a significantly higher prostaglandin level compared to subjects who didn’t ^16^.

Membrane sweeping was found to be a safe method of induction with no increased risk of maternal and neonatal adverse outcomes, lower post-mature pregnancy rates and reduced formal pharmacological inductions ^17, 18^. It increased spontaneous delivery rate, reduced oxytotic use, shortened induction to delivery interval and increased patient satisfaction ^19^. It can be utilized safely to induce labour at 41 weeks which makes it particularly useful in a low resource setting ^20^. Vaginal birth after caesarian section was shown to end successfully even without the use of pharmacological induction and augmentation when induced with membrane sweeping ^21^. Membrane sweeping shortened the cervix as measured ultrasonically and it was an independent predictor of reduction of caesarean deliveries ^22^. Additionally, when used in GBS positive mothers it did not affect the adequacy of antibiotic treatment ^23^.

In some studies membrane sweeping was not effective and had certain risks. Foong et al found that while membrane sweeping has beneficial effects on labour and delivery, these appeared to be limited to nullipara with unfavorable cervices ^24^. Micah et al showed that membrane sweeping done beyond 1cm dilated cervix may be associated with a higher risk of pre-labor rupture of membranes ^25^. While some studies conclude membrane sweep as a safe and effective method of induction in vaginal birth after caesarean section, some show no significant effect on onset of labour, duration of pregnancy, rate of formal induction and rate of repeat caesarean deliveries ^26^. A few studies show that membrane sweep is painful ^22, 27^.

Membrane sweeping has been compared and analyzed against pharmacological methods of induction with regards to effectiveness and safety by Everette et al, in 1999. They showed that serial sweeping of membranes is more effective than single dose dinoprostone induction with one-fourth the cost and a lower risk of post-date inductions ^28^.

NICE guideline on inducing labour recommends membrane sweep and cervical massage as adjuncts to formal induction and not as primary induction methods^6^.

# CHAPTER 3 – Objectives and aims

1. Overall objectives
   1. To determine the percentage of women going into spontaneous labour after membrane sweep and cervical massage.
   2. To determine the survival (without spontaneous labour) after membrane sweep and cervical massage.
   3. To determine the maternal acceptability of membrane sweep and cervical massage
2. Specific objectives
   1. To compare labour outcomes after each intervention in terms of,
   2. Emergency Caesarean delivery rates after spontaneous labour following membrane sweep and cervical massage.
   3. Percentage of Oxytocin augmentation during labour following membrane sweep and cervical massage.
   4. Uterine hyperstimulation rates following membrane sweep and cervical massage.
3. To compare neonatal outcome measures in term of,
4. APGAR at 5 minutes following membrane sweep and cervical massage.
5. Rate of APGAR <7 at 5 minutes following membrane sweep and cervical massage.
6. To compare maternal outcome with regards to,
7. Postpartum bleeding rates following membrane sweep and cervical massage.
8. Maternal pyrexia rates following membrane sweep and cervical massage.
9. Duration of hospital stay following membrane sweep and cervical massage.

# CHAPTER 4 – Research plan

1. Study design

This study will be a prospective single blinded randomized controlled clinical trial. The study participants will be followed up from administration of the mechanical method of induction until 24 hours after delivery and the baby will be followed up until 5 minutes post-partum.

1. Study location

This study will be conducted at a tertiary care teaching hospital

1. Study duration

This study will continue from February 2020 to July 2020.

1. Study null hypothesis

These is no statistically significant difference between the effectiveness of cervical massage and membrane sweep to ripen the cervix, to establish spontaneous labour and prevent formal induction of labour.

There is no statistically significant difference between the maternal acceptability about the use of membrane sweep and cervical massage to ripen the cervix.

1. Study procedure
   1. Due ethical clearance will be obtained from the ethical review committee of the Faculty of Medicine, University of Colombo.
   2. Permission from the Director of the tertiary care hospital to conduct the study in specified antenatal wards will be obtained in writing.
   3. Pregnant women with an uncomplicated antenatal period without any indication for early delivery, who meet the inclusion criteria and exclusion criteria will be recruited during the 38^th^ week of gestation to the study following informed consent.
   4. Informed written consent will be obtained by the principal investigator at recruitment (during 38^th^ week of gestation) to the study and before enrollment. Informed written consent will be obtained after clear explanation of the study design, procedure, purpose, benefits and risks. Information sheets will be available in Sinhala, Tamil and English languages. Consent form will be available in Sinhala Tamil and English languages for clarity and comprehension. (Annexure A – Sinhala version of the consent form)

(Annexure B – Tamil version of the consent form)

(Annexure D – English version of the consent form)

(Annexure E – Sinhala version of the information sheet)

(Annexure F – Tamil version of the information sheet)

(Annexure G – English version of the information sheet)

Prospective participants will be given 6 days to read the information sheets, discuss with family, inquire and clarify any doubts from the investigators. The information sheets will contain contact details of investigators for use of the participants. (Dr Sameera Madugalle -0776748181). Participants will have access to investigators over the phone at any time and they will be able to visit the hospital during routine working hours. At 39^th^ week of gestation participants will be interviewed by the investigators. During this interview, participant’s understanding of information provided will be assessed by following direct questions^31^.

- - 1. Describe in your own words what will be done to you.
    2. Describe what you consider to be the major risks associated with this procedure.
  1. A data collection form will be opened for each study participant. General information excluding personal details will be obtained from study participants. Personal details will not be obtained since the patients will not be followed up with regards to the study afterwards (Annexure C – Data collection form).
  2. Eligible study participants will be allocated randomly to three study groups.
     1. Group MS – Induction of labour with membrane sweeping
     2. Group CM – Induction of labour with cervical massage
     3. Group C – Neither membrane sweeping nor cervical massage / No intervention
  3. Research Randomizer available at https://www.randomizer.org/ will be used to generate a set of random numbers. Equal sized cards will be prepared and a random number with its allocated method of induction will be printed on each card. These cards will be kept locked in an opaque container in the ward. After a study participant signs the consent form, the next available sequential number will be extracted from the said container and the method mentioned will be administered as the method of induction of labour.
  4. This being single blind randomized controlled study, except for the study participants, staff and investigators will know the method of induction of each participant. All three arms will be considered as receiving equal treatment. As both methods are mechanical, the risks of uterine rupture do not differ. While individual studies have revealed the efficacy of both methods, there are no comparative studies between the two methods.
  5. The membrane sweep or the cervical massage will be performed once at 39^th^ and once at 40^th^ weeks of gestation if spontaneous labour does not follow. Pre-induction clinical assessment to ensure maternal well-being, CTG, ultrasound scan to confirm fetal wellbeing will be performed.
  6. Modified Bishops’ Score will be assessed prior to induction. The possibility to insert the examining finger through the cervical canal will be assessed to ensure all three interventions will be equally applicable as per inclusion criteria.
  7. In Group MS, membrane sweep will consist of insertion of one finger through the internal OS and performing three circumferential passes separating the chorio-amnion from the lower uterine segment.
  8. In Group CM, cervical massage consists of three circumferential passes of around the cervix massaging the cervix with examiner’s index and middle fingers for 15-30 seconds.
  9. In Group C, none of the above methods will be used and theses women will be used as the control group for the study.
  10. Repeat CTG will be performed 2 hours after intervention in each group.
  11. Uterine contractions, maternal body temperature, pulse rate, blood pressure, and fetal heart rate will be monitored every 4 hours for 24 hours after each intervention.
  12. These women will be followed up every 3^rd^ day from intervention until 24 hours after delivery or till 40+6 weeks of gestation. They will be reviewed at 40+6 weeks of gestation.
  13. Modified Bishop’s Score will be calculated for each woman.
      1. If the Modified Bishops’ Score is 7≤ and labour will be allowed to progress. These women will be followed up according to protocol.
      2. If Modified Bishops’ Score remains 6≥, it will be considered as a failure of cervical ripening and the ward obstetrician will decide on further plan of management. These women will be followed up until discharge after delivery.
  14. Modified Bishops’ Score will be assessed if the study participant complains of pain and is found to have 2≤ moderate contractions (A contraction lasting for 20-40 seconds) per 10 minutes or more ^1^. If the Modified Bishops’ Score is found to be 7≤, the time of assessment will be noted and the labour will be allowed to progress.
  15. Management of labour once established will be conducted according to national guidelines. Amniotomy and oxytocin augmentation will be used to correct any poor progression of labour.
  16. Data will be collected during the duration of the study using designed data collection forms.
  17. Statistical analysis will be conducted using Statistical Package for Social Sciences Version 19.
  18. Results will be published according to pre-determined dissemination protocols.

1. Flow diagram of study

Follow up till 40+6, Assessment at 40 +6 shows failure of cervical ripening

Formal induction with mechanical or pharmacological methods

1. Study instruments
   1. Data collection form will consist of following sections.

(Annexure G – Sinhala version of data collection sheet)

(Annexure H – English version of data collection sheet)

(Annexure I – Tamil version of data collection sheet)

- - 1. Section A - General details

Age, Gravidity, Parity, Period of amenorrhea, Maternal height, Weight, Body mass index, Past pregnancies, Past modes of delivery, Indication for induction

- - 1. Section B – Progress of cervical ripening

Modified Bishop’s Score at 39^th^ week of gestation, Modified Bishop’s Score at 40^th^ week of gestation, Modified Bishop’s Score at 40+6 week of gestation

- - 1. Section C – Delivery details

Mode of delivery, Time taken into labour suite, Time of delivery, Total duration of labour, oxytocin augmentation, maximum contraction rate, estimated blood loss, APGAR score at 5 minutes.

- - 1. Section D – Maternal Acceptability

Assessment of maternal acceptability on mode of induction including its complications and outcomes.

- 1. Membrane sweep

Membrane sweep will involve insertion of the operator’s finger through the internal OS of cervix followed by three 360^0^ circumferential passes separating the chorio-amnion from the lower segment of the uterus over 15-30 seconds. Membrane sweep is associated with increased discomfort and per vaginal bleeding ^26^.

- 1. Cervical massage

Cervical massage consists of three circumferential passes around the cervix massaging the cervix with examiner’s index and middle fingers for 15-30 seconds.

- 1. Modified Bishops’ Score

Modified Bishops’ Score is a set of measurements taken during the vaginal examination. A score of 7≤ will be considered favorable.

| Parameter | Score | | | |
| --- | --- | --- | --- | --- |
|  | 0 | 1 | 2 | 3 |
| Station | -3 | -2 | -1/0 | +1≤ |
| Dilatation | 0-1 | 1-2 | 3-4 | 5≤ |
| Cervical length | >3 cm | 3-2 cm | 2-1 cm | 1-0 cm |
| Consistency | Firm | Medium | Soft |  |
| Position | Posterior | Axial | Anterior |  |
| Modifiers | | | | |
| If there is pre-eclampsia Add one point  Each per previous vaginal delivery  If it is a post-dates pregnancy  If nulliparous Deduct one  If there is pre-term pre-labor rupture of membranes point | | | | |

1. Procedures and treatments undertaken during the study
   1. Membrane sweep
   2. Cervical massage
   3. Abdominal and vaginal examination to assess the maternal, fetal well-being and the progress of labour.
   4. Intrapartum continuous electronic fetal monitoring
   5. Oxytocin augmentation in case of suboptimal uterine contractions.
   6. Instrument delivery or emergency caesarian section if indicated
2. Selection and withdrawal of study participants
   1. Sample population

Pregnant women with an uncomplicated antenatal period without any indication for early delivery, who meet the inclusion criteria and exclusion criteria will be recruited during the 38^th^ week of gestation to the study following informed consent.

- 1. Inclusion criteria

Recruited women will,

- - 1. 38-40 completed weeks of gestation,
    2. No clear indication for early delivery, emergency, or elective cesarean delivery
    3. Had normal fetal heart rate rhythm on CTG, normal fetal dopplers on ultrasound scan, adequate growth for gestational age,
    4. Modified Bishops’ Score ≤6, but the examining finger was admissible into the cervix during pelvic examination, so all interventions were equally applicable,
  1. Exclusion criteria

Women will not be selected for recruitment if,

- - 1. Intra uterine fetal demise,
    2. Ruptured membranes
    3. Abnormal amniotic fluid index,
    4. Fetal anomalies
    5. Abnormal fetal growth – growth restriction or macrosomia
    6. Any contraindication for induction of labor
    7. Latex allergies (Latex gloves were worn during certain procedures during the study).
    8. Tightly closed cervix where membrane sweep would not be possible
  1. Criteria for termination of study
     1. The study will be terminated if these mechanical methods of cervical ripening impose unreasonable and statistically significant risk. These risks include uterine rupture, cervical injuries, maternal infections and neonatal injury. If these complications occur at a statistically significant level the study will be terminated.
  2. Participant withdrawal
     1. Study participants may withdraw previously given consent and withdraw from the study at any moment during the study period. In case of such voluntary withdrawal, participants will be advised to inform the study team immediately after the decision to withdraw has been made. This is to ensure that the data obtained will be destroyed and not included in statistical analysis.
     2. Participants will be withdrawn from the study if severe Latex allergies, vasovagal attacks, cervical injuries, uterine rupture or maternal infections occur.
     3. Recruited participants will be removed from the study if they go into active labour (Achieve a Modified Bishops’ Score 7≤) before the cervical ripening in undertaken.
     4. Recruited participants will be withdrawn from the study if they develop an indication for immediate delivery before the cervical ripening is undertaken.
     5. Withdrawn participants will be managed according to the routine ward protocol.
     6. Whenever a participant withdraws from the study, their data sheet will be destroyed and will not be used in the analysis.
     7. To complete the sample size requirements, further participants will be recruited to replace the withdrawn individuals as needed.
  3. Safety concerns during the study period
     1. Study participants will be briefed about the risk of complications during the study before informed consent is obtained. Risks include latex allergies, vaso-vagal attacks, cervical injuries, uterine rupture and maternal infections.
     2. Prevalence of latex allergy id 4.9% in general population^34^. Total prevalence of vasovagal syncope is about 2% and it’s rare during cervical procedures^35^. Significant cervical injury is uncommon in membrane sweeping. There is no significant increase in maternal or neonatal infection after membrane sweep^36^.
     3. The entire procedure, including the investigated procedures are a part of routine obstetric practice. Therefore, the study participants will not be placed under additional risk for the sake of the study.
     4. All procedures will be done under appropriate universal precautions for prevention of infections, which includes wearing sterile latex gloves.
     5. If latex allergy is identified, steroids and antihistamines will be administered as indicated as rescue medications, vitals will be monitored closely for 24 hours. If severe allergic reactions or anaphylaxis occur the participant will be withdrawn from the study.
     6. If vasovagal attack occurs, no further cervical massage or membrane sweeps will be performed on the participant, the participant will be withdrawn from the study, vasovagal attack will be managed as indicated with close monitoring of the participant.
     7. If a cervical tear occurs, immediate examination and repair will be undertaken, and the participant will be closely monitored. The participant will be removed from the study.
     8. If uterine rupture occurs, emergency exploratory laparotomy will be performed and managed as need arises. Uterine primary repair or hysterectomy may be warranted as indicated. Blood transfusions may also be necessary.
     9. If maternal infections set in, basic investigation including cultures will be performed, if unstable, the participant will be managed in HDU or ICU and broad-spectrum antibiotics will be administered following microbiology team review.
     10. If any psychological concerns arise relevant referrals will be made, and a care plan will be agreed upon.
     11. In case of any concern participants will be advised to visit unit clinics for follow-up care after the study.
     12. In case of any adverse event mentioned above or unexpected adverse event occurring, the Ethical review committee and all the participants will be informed immediately. If requested by the ERC a re-consenting will be conducted.
  4. Benefits to participants

Adjuncts for induction (membrane sweep or cervical massage) can be used to prevent formal induction with all its inherent risks (Number needed to treat – 8) and to reduce induction to delivery time.

1. Sample size calculation
   1. Based on an earlier study, which reported an increase in the spontaneous vaginal delivery rate from 75% to 90% with sweeping, sample size calculation using an Alfa of 0.05 and Beta of 0.8 indicated that 100 women were needed in each group for an appropriately powered randomized study on the effect of membrane sweeping in conjunction with formal labor induction. Therefore, a total of 300 women will be recruited into the study.


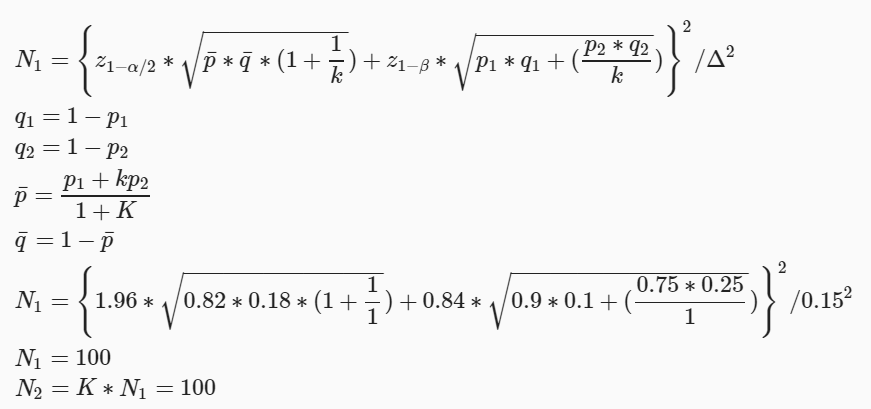


1. Outcome measures
   1. Overall outcome measures
      1. Percentage of women going into spontaneous labour after membrane sweep and cervical massage.
      2. Survival (without spontaneous labour) after membrane sweep and cervical massage.
      3. Maternal acceptability of membrane sweep and cervical massage
   2. Specific outcome measures – Delivery outcomes
      1. Emergency Cesarean delivery rates after spontaneous labour following membrane sweep and cervical massage.
      2. Percentage of Oxytocin augmentation during labour following membrane sweep and cervical massage.
      3. Uterine hyperstimulation rates following membrane sweep and cervical massage.
   3. Specific outcome measures - Maternal outcomes
      1. Postpartum bleeding rates following membrane sweep and cervical massage.
      2. Maternal pyrexia rates following membrane sweep and cervical massage.
      3. Duration of hospital stay following membrane sweep and cervical massage.
   4. Specific outcome measures – Neonatal outcomes
      1. APGAR at 5 minutes following membrane sweep and cervical massage.
      2. Rate of APGAR <7 at 5 minutes following membrane sweep and cervical massage
2. Data safety monitoring plan
   1. Data Safety Monitoring Board (DSMB)

Entire procedure, including the investigated procedures are a part of routine obstetric practice with minimal risk to patient. Therefore, the study participants will not be placed under additional risk for the sake of the study^32^.

Therefore, Data Safety Monitoring Board will comprise of the two external subject-experts and an external biostatistician. (Short CV’s attached – Annexures J, K, L)

- 1. Oversight responsibilities

Oversight of the trial is provided by the principle investigator and co-investigators.

- 1. Aspects reviewed by the Data Safety Monitoring board

Following aspects will be reviewed and written reports.

- - 1. Recruitment of participants
    2. Adverse events
    3. Data completeness
    4. Outcome data
    5. Protocol non-compliance
    6. New and relevant information obtained
  1. Monitoring procedure

The DSMB assures that informed consent is obtained prior to performing any research procedures, that all subjects meet eligibility criteria, and that the study is conducted according to the IRB-approved research plan.

Study data are always accessible for the DSMB to review. The DSMB reviews study conduct on a 3-monthly basis. The DSMB reviews adverse events individually real-time and in aggregate on a 3-monthly basis. The DSMB reviews serious adverse events in real-time. The DSMB ensures all protocol deviations, adverse events, and serious adverse events are reported to the ERC every 3 months, according to the applicable regulatory requirements.

- 1. Collection and reporting of adverse events and serious adverse events

For this study, the following standard AE definitions are used:

- - 1. Adverse event: Any unfavorable and unintended sign (including an abnormal laboratory finding), symptom, or disease temporally associated with the use of a medical treatment or procedure, regardless of whether it is considered related to the medical treatment or procedure.
    2. Serious Adverse Event: Any AE that results in any of the following outcomes:
       1. Death
       2. Life-threatening Event requiring inpatient hospitalization or prolongation of existing hospitalization
       3. Persistent or significant disability/incapacity

AEs are graded according to the following scale

1. Mild: An experience that is transient and requires no special treatment or intervention. The experience does not generally interfere with usual daily activities. This includes transient laboratory test alterations.
2. Moderate: An experience that is alleviated with simple therapeutic treatments. The experience impacts usual daily activities. Includes laboratory test alterations indicating injury, but without long-term risk.
3. Severe: An experience that requires therapeutic intervention. The experience interrupts usual daily activities. If hospitalization (or prolongation of hospitalization) is required for treatment it becomes an Serious Adverse Event.

The study uses the following AE attribution scale

1. Not related: The AE is clearly not related to the study procedures (i.e., another cause of the event is most plausible, and/or a clinically plausible temporal sequence is inconsistent with the onset of the event).
2. Possibly related: An event that follows a reasonable temporal sequence from the initiation of study procedures, but that could readily have been produced by a number of other factors.
3. Related: The AE is clearly related to the study procedures.

AEs are identified during clinical monitoring following intervention as detailed in the protocol.

SAEs and specific procedure-associated AEs are reported to the ERC within 24 hours. In addition, all AEs are reported according to the ERC every 3 months.

- 1. Management of risks to subjects
     1. Study participants will be briefed about the risk of complications during the study before informed consent is obtained. Risks include latex allergies, vaso-vagal attacks, cervical injuries, uterine rupture and maternal infections.
     2. Prevalence of latex allergy if 4.9% in general population^34^. Total prevalence of vasovagal syncope is about 2% and its rare during cervical procedures^35^. Significant cervical injury is uncommon in membrane sweeping. There is no significant increase in maternal or neonatal infection after membrane sweep^36^.
     3. The entire procedure, including the investigated procedures are a part of routine obstetric practice. Therefore, the study participants will not be placed under additional risk for the sake of the study.
     4. If latex allergy is identified, steroids and antihistamines will be administered as indicated as rescue medications, vitals will be monitored closely for 24 hours. If severe allergic reactions or anaphylaxis occur the participant will be withdrawn from the study.
     5. If vasovagal attack occurs, no further cervical massage or membrane sweeps will be performed on the participant, the participant will be withdrawn from the study, vasovagal attack will be managed as indicated with close monitoring of the participant.
     6. If a cervical tear occurs, immediate examination and repair will be undertaken, and the participant will be closely monitored. Participants will be removed from the study.
     7. If uterine rupture occurs, emergency exploratory laparotomy will be performed and managed as need arises. Uterine primary repair or hysterectomy may be warranted as indicated. Blood transfusions may also be necessary.
     8. If maternal infections set in, basic investigation including cultures will be performed, if unstable, the participant will be managed in HDU or ICU and broad-spectrum antibiotics will be administered following microbiology team review.
     9. If any psychological concerns arise relevant referrals will be made, and a care plan will be agreed upon.
     10. In case of any concern participants will be advised to visit unit clinics for follow-up care after the study.
     11. In case of any adverse event mentioned above or unexpected adverse event occurring, the Ethical review committee and all the participants will be informed immediately. If requested by the ERC a re-consenting will be conducted.
     12. Criteria for termination of study

The study will be terminated if these mechanical methods of cervical ripening impose unreasonable and statistically significant risk. These risks include uterine rupture, cervical injuries, maternal infections, and neonatal injury. If these complications occur at a statistically significant level the study will be terminated.

- 1. Plan for analysis of safety

All adverse events aggregate, and all serious adverse events individually will be analyzed.

- 1. Plan for data management

Compliance of regulatory documents and study data accuracy and completeness will be maintained through an internal study team quality assurance process.

1. Statistical analysis
   1. All statistical analyses will be performed with the Statistical Package for Social Sciences for Windows.
2. Financial arrangements
   1. The study is self-funded.
   2. Compensation, re-imbursement, incentives for study participants
      1. Participation will be purely voluntary with full understanding of the minimal risks associated with mechanical induction of labour and there will be no incentives for participation or financial compensation for any injury related to the conduct of the study.
3. Publication policy
   1. The identity of study participants will not be revealed when publishing study results. Papers based on the study will be published in Local, National and International scientific meetings and journals as posters, video-presentations, oral presentations and articles. Study results will be made available to all the study participants.
4. Ethical considerations
   1. Autonomy

Informed written consent will be obtained by the principle investigator at recruitment (during 38^th^ week of gestation) to the study and before enrollment. Participants will be given clear information on the study purpose, design, possible risks, benefits, outcome and the right to withdraw from the study at any time even after providing consent initially. Information sheets will be available in Sinhala, Tamil and English languages. Consent form will be available in Sinhala Tamil and English languages for clarity and comprehension.

(Annexure A – Sinhala version of the consent form)

(Annexure B – Tamil version of the consent form)

(Annexure D – English version of the consent form)

(Annexure E – Sinhala version of the information sheet)

(Annexure F – Tamil version of the information sheet)

(Annexure G – English version of the information sheet)

Prospective participants will be given 6 days to read the information sheets, discuss with family, inquire and clarify any doubts from the investigators. Information sheets will contain contact details of investigators for use of the participants. At 39^th^ week of gestation participants will be interviewed by the investigators. During this interview, participant’s understanding of information provided will be assessed by following direct questions^31^.

- - 1. Describe in your own words what will be done to you.
    2. Describe what you consider to be the major risks associated with this procedure.
  1. Beneficence and Non-maleficence

Safety of participants will be ensured by having clearly defined protocols for managing possible risks.

Since all the procedures are a part of routine obstetric practice the study participant will not be placed at additional risk for the sake of the study.

Formal induction of labour with mechanical and pharmacological agents carry a significant risk of uterine hyperstimulation, fetal distress, uterine rupture and operative deliveries. Cervical ripening has been shown to reduce the need for formal induction thereby reducing above risks. Study participants will have the clear direct benefit of not facing risks of formal induction.

By following clearly defined inclusion, exclusion and withdrawal criteria the safety of the study participants will be ensured.

Study participants will be comprehensively monitored during the study to ensure maternal and fetal safety and to detect any adverse events as early as possible.

Participants will be managed according to ward protocols if ever they withdraw from the study.

Participants will be briefed about the staff and investigators with direct access to patient records and the study records will be kept under lock and key until the completion of the study and will be destroyed by shredding to protect confidentiality.

The data sheets will be handled only by the investigators and will be kept under lock and key while not in use. During publication, identity of participants will not be revealed. Following completion of the trial all anonymized study data and the study protocol will be made available for free public access in a data repository. Medical records (Bed Head Ticket) will be stored at the hospital as per routine regulations and the investigators will only keep anonymized study data.

Equipoise

As investigators are unaware of the study outcome, all the study participants will be treated equally.

- 1. Justice

Safety of study participants will not be compromised in view of doing good for the future patients who probably will undergo the management decisions based from the study.

1. No conflicts of interest declared.
2. Work plan and timelines

| Month/  year | Submission of research proposal | BOS approval | Ethical clearance | Data collection | Data analysis | Final publication |
| --- | --- | --- | --- | --- | --- | --- |
| May 2019 |  |  |  |  |  |  |
| June 2019 – Jan 2020 |  |  |  |  |  |  |
| Feb 2020 |  |  |  |  |  |  |
| March 2020 |  |  |  |  |  |  |
| April 2020 |  |  |  |  |  |  |
| May 2020 |  |  |  |  |  |  |
| June 2020 |  |  |  |  |  |  |
| July 2020 |  |  |  |  |  |  |
| Aug 2020 |  |  |  |  |  |  |
| Sept 2020 |  |  |  |  |  |  |
| Oct 2020 |  |  |  |  |  |  |

# CHAPTER 5 – Annexures

Annexure A – Sinhala version of information sheet

Membrane sweep සහ Cervical massage මඟින් ගැබ් ගෙල දරු ප්‍රසූතියක් සඳහා සූදානම් කිරීමේ සාර්ථකත්වය සහ මාතෘ තෘප්තිමත්භාවය සසඳා බැලීම

**තොරතුරු පත්‍රිකාව**

වෛද්‍ය ටී.එම්.එස්.එස්.බී. මඩුගල්ලේ වන මම, කොළඹ ද සොයිසා කාන්තා රෝහලේ ප්‍රසව හා නාරිවේද වෛද්‍යවරයෙක්මි. ඔබට, අප විසින් කරනු ලබන, දරු ප්‍රසූතියකට ගැබ් ගෙල සූදානම් කරන ක්‍රම දෙකක කාර්යක්ෂමතාව හා මාතෘ තෘප්තිමත් බව සසඳා බැලීමේ පර්යේෂණයකට සහභාගි වන ලෙස කාරුණිකව ඉල්ලා සිටිමි. මෙම පර්යේෂණය ඔබ ඇතුලත් වී සිටින විටදී වාට්ටුවේ දී සිදු කෙරෙනු ඇත.

1. **අරමුණු**

මෙම පර්යේෂණයේ ප්‍රධාන අරමුණ වනුයේ සාමාන්‍ය දරු ප්‍රසූතියක් සඳහා ගැබ් ගෙල සූදානම් කිරීමේ ක්‍රම දෙකක් සසඳා බැලීමයි. මෙහි දී එම ක්‍රම දෙකට අදාල ව,

- සාමාන්‍ය දරු ප්‍රසූතියකට යන මව්වරු ගණන
- ක්‍රමයන් අසාර්ථක වීමේ සම්භාවිතාව, (Failure rate)
- දරු ප්‍රසූතිය දක්වා ගත වන කාලය, (Induction to delivery time)
  1. සාමාන්‍ය දරු ප්‍රසූතියක ප්‍රතිශතය සති (Vaginal delivery rates)
- සිසේරියන් සැත්කම් මගින් සිදුවන උපත් ප්‍රතිශතය, (Caesarian section rate)
- ප්‍රසූතිය තුල දී ඔක්සිටොසින් භාවිතා වන ප්‍රතිශතය, (Oxytocin augmentation rate)
- අධික ගර්භාශ සංකෝචනය, (uterine hyperstimulation)
- ගර්භාශය තුවාල වීම, (uterine rupture)
- උණ, (maternal fever)
- රෝහල්ගතව සිටීමට සිදු වන කාලය , (Duration of hospital stay)
- ළදරුවාගේ සෞඛ්‍ය තත්වය
- මවක ලෙස එම ක්‍රම ගැන ඔබේ තෘප්තිමත් බව

යන කරුණු සසඳා බැලේ.

1. **සහභාගිත්වය පූර්ණ නිදහස් තීරණයකි**

මෙම පර්යේෂණයට සහභාගි වීම හෝ නොවීමේ තීරණය ඔබේ අයිතියකි. ඔබ මේ අවස්ථාවේ කැමැත්ත ලබා දුන්නද පසුව එයින් ඉවත් වීමේ හැකියාව ද ඔබට ඇත.

1. **පර්යේෂණය සිදු කෙරෙන පිලිවෙල**

තෝරාගත් මව්වරු අහඹු ලෙස කණ්ඩායම් 3 කට වෙන් කෙරෙනු ඇත. පළමු කණ්ඩායමට Membrane sweep ද, දෙවන කණ්ඩායමට Cervical massage ද සිදු කෙරෙනු ඇත. තුන් වන කණ්ඩායම පාලන කණ්ඩායම (Control group) නිසා ඉහත ක්‍රම දෙකම යොදා ගනු නොලැබේ. ගැබ් ගෙල ප්‍රසූතියට සූදානම් කිරීමේ ක්‍රම දෙක Membrane sweep හා Cervical massage වේ. Membrane sweep යනු PV පරීක්ෂාවක් හරහා වෛද්‍යවරයාගේ ඇඟිල්ලකින් දරුවාගේ ගර්භ පටලය වෙන් කිරීමයි. මෙහි දී ඔබට මඳ වේදනාවක් දැනිය හැකි අතර පසුව සුලු රුධිර වහනයක් ඇති විය හැක. Cervical massage යනු PV පරීක්ෂණයක් හරහා වෛද්‍යවරයාගේ ඇඟිල්ලකින් ඔබගේ ගැබ් ගෙල උත්තේජනය කිරීමයි. මෙහි දී ඔබට මඳ වේදනාවක් දැනිය හැකියි. ගර්භණී මව්වරුන්ට සති 39 දී හා ප්‍රසූතිය සිදු නොවුනහොත් සති 40 දී මේ ගැබ් ගෙල සූදානම් කිරීමේ ක්‍රමය සිදු කෙරෙනු ඇත. ඉන් පසු ඔබව දරුවා ලැබ නිවස බලා යනතෙක්ම පරීක්ෂා කෙරෙනු ඇත. ඔබට ගර්භාෂ සංකෝචනයන් (මධ්‍යම ප්‍රමාණයේ) 2ක් එන විට ඔබේ ගැබ් ගෙල නැවත පරීක්ෂා කෙරෙනු ලැබේ. ඔබේ ගැබ් ගෙල ප්‍රසූතියකට නුසුදුසු නම් ඔබට සම්මත ක්‍රමයට අනුකූල ව ප්‍රතිකාර කර ඔබව නැවත ප්‍රසූත වේදනාව සමඟ පරීක්ෂා කෙරෙනු ඇත. ඔබේ ගැබ් ගෙල ප්‍රසූතියකට සුදුසු නම් ඔබව සූතිකාගාරයට ගෙන ඔබේ දරු ප්‍රසූතිය ඉදිර්යට යාමට ඉඩ සලස්වනු ලැබේ. යම් හෙයකින් බ සති 40 යි දින 6ක් වන තුරු ද ප්‍රසූතියකට නොගියහොත් ඔබව නැවත් පරීක්ෂා කෙරෙනු ඇති. ඒ අනුව විශේෂඥ වෛද්‍යවරයා වෙනත් ක්‍රමයකින් ඔබේ ගැබ් ගෙල සූදානම් කිරීමට තීරණය කරනු ඇත. ඉන් පසු ඔබව දරුවා ලැබ නිවසට යන තුරුම පරීක්ෂා කෙරෙනු ඇත.

1. **මෙම පර්යේෂණයෙන් ඔබට හා වෙනත් මවුවරුන්ට ඇති වෙන වාසි**

Prostaglandin හෝ Foley catheter ක්‍රම මඟින් ගැබ් ගෙල සූදානම් කිරීමේ දී අධික ගර්භාෂ සංකෝචනය, ගර්භාෂයට තුවාල වීම, දරුවාගේ හෘද ස්පන්දනය වැඩිවීම හා සැත්කම් මඟින් දරු ප්‍රසූතියේ ප්‍රවණතාවය වැඩි වේ. Membrane sweep හා cervical massage මඟින් ගැබ් ගෙල සූදානම් කිරීම මඟින් අනෙකුත් සම්මත ක්‍රම මඟහැරවීම නිසා ඒ හා බැඳුනු අවදානම් තත්වයන් මඟහැරේ. මෙම පර්යේෂණයට සහභාගි වීම මඟින් එම අවදානම් තත්වයන් මඟ හැරීමේ පැහැදිලි සෘජු වාසිය ලැබේ. ඔබට මෙන් ම අනාගතයේ බොහෝ මවුවරුන්ට මෙයින් යහපතක් වෙනු ඇතැයි අපේ බලාපොරොත්තුවයි.

1. **මෙම පර්යේෂණයේ දී කලාතුරකින් ඇති විය හැකි සංකූලතා**

සංකූලතා ලෙස අත්වැසුම් නිමවා ඇති ලේටෙක්ස් වලට අසාත්මිකතාව, හෘද ස්පන්දනය හා රුධිර පීඩනය අඩු වීම, ගර්භාෂයට හෝ ගැබ් ගෙලට තුවාල වීම හා විෂබීජ ශරීරගත වීම දැක්විය හැකියි.

මෙම පර්යේෂණයේදී සිදු කෙරෙන් සියලු ක්‍රියාවලියන් එදිනෙදා ප්‍රතිකාරවලදී කෙරෙන දේවල් වන අතර එනිසාම පර්යේෂණයට සහභාගි වූ පමණින් කිසිදු අමතර අන්තරායකට ඔබට මුහුණ දීමට සිදු නොවෙනු ඇත.

- 1. ඔබට අසාත්මිකතාවක් ඇති වුවහොත් ඔබට ඒ සඳහා ප්‍රතිකාර කර, වාට්ටුවේ පැය 24ක් රඳවා තබා ගෙන ඔබ පිළිබඳ පරීක්ෂණ කෙරෙනු ඇත. අසාත්මිකතාව දරුණු එකක් නම් ඔබව පර්යේෂණයෙන් ඉවත් කරනු ඇත.
  2. ඔබේ හෘද ස්පන්දනය හෝ රුධිර පීඩනය අඩු වුවහොත් ඔබට ඒ සඳහා ප්‍රතිකාර කර, ඔබව පර්යේෂණයෙන් ඉවත් කරනු ලැබේ. තව දුරටත් ගැබ් ගෙල සූදානම් කිරීමේ ක්‍රම ඔබ සම්බන්ධව යොදා නොගනු ඇත.
  3. ඔබේ ගැබ් ගෙලට තුවාල සිදු වුවහොත් එය වහාම පිලිසකර කෙරෙනු ඇත.
  4. ඔබේ ගර්භාෂයට හානි වුවහොත් වහාම සැත්කමකට ඔබව ලක් කෙරෙනු ඇත. හැකි සැම විටම ගර්භාෂය අලුත්වැඩියා කරන අතර අත්‍යවශ්‍ය නම් පමණක් ගර්භාෂය ඉවත් කිරීමට කටයුතු කෙරෙනු ඇත.
  5. ඔබේ ශරීරයට විෂබීජයක් ඇතුලු වුවහොත්, ඔබට ප්‍රතිජීවක ඖෂධ ලබ දෙනු ඇත. ඒ සම්බන්ධ විශේෂඥ වෛද්‍යවරුන් ඔබේ ප්‍රතිකාර සඳහා සහභාගි කර ගනු ඇත.
  6. මානසික ගැටළු වලදී අදාල වෛද්‍යවරුන් හරහා ඔබට අවශ්‍ය වෛද්‍ය ප්‍රතිකාර නොපමා ව ලබා දෙනු ලැබේ.

පර්යේෂණයෙන් පසුව පවා ඔබට අවශ්‍ය විටක අපගේ සායන වෙත පැමිණීමේ හැකියාව ඇත.

ඉහත සඳහන් සංකූලතා හෝ වෙනත් අනපේක්ෂිත සංකූලත ඇති වුවහොත් ඒ බව අදාල බලධාරීන්ට දන්වනු ලබන අතර අවශ්‍ය නම් ඔබ සමඟ නැවත සාකච්ඡා කෙරෙනු ඇත.

1. **ගෙවීම්, වන්දි ගෙවීම්, දීමනා**

මෙම පර්යේෂණයට සහභාගි වීම සම්පූර්නයෙන් ම ස්වේච්ඡාවෙන් සිදු වේ. මෙම පර්යේෂණ්යේ දී ඇති විය හැකි වාසි හා අවදානම් තත්වයන් ගැන පැහැදිලි අවබෝධයකින් යුතුව සිදූ වේ. මේ සඳහා කිසිදු ගෙවීමක් හෝ අනපේක්ෂිත අතුරු ආබාධ සඳහා වන්දි ගෙවීමක් සිදු නොවනු ඇති. එහෙත් එබඳු අවස්ථාවක දී අවශ්‍ය සියලු ප්‍රතිකර්ම ලබා දෙනු ඇත.

1. **රහසිගත බව**

ඔබේ ජීව දත්ත පර්යේෂකයින්ගෙන් හා පර්යේෂණ පාලන ආයතනයන්ගෙන් එපිට කිසි සේත් ලබා නොදෙනු ඇත. පර්යේෂණය අවසානයේ ඔබේ ඇඳ ඉහ පත් රෝහල තුල සාමාන්‍ය පරිදි තැන්පත් කෙරෙන අතර, පර්යේෂණයට අදාල දත්ත පමණක් වසර 3 ක කාලයක් තුල පර්යේෂණ කණ්ඩායම විසින් ලඟ තබා ගනු ලැබේ. ඔබ හඳුනා ගත් හැකි කිසිම තොරතුරක් එම දත්ත වල අඩංගු නොවනු ඇත. එම වසර තුන අවසානයේ එම දත්ත පවා විනාශ කෙරෙනු ඇත.

1. **පර්යේෂණයෙන් ඔබට ඉවත් විය හැකි හා ඉවත් කරනු ලබන අවස්ථා**

යම් හෙයකින්, ඖෂධීය ක්‍රම මඟින් ගැබ් ගෙල ප්‍රසූතියට සූදානම් කිරීමේ දී ඇති විය හැකි සංකූලතා වන ගර්භාශය තුවාල වීම, ගැබ් ගෙල තුවාල වීම, විෂබීජ ශරීර ගත වීම හෝ ළදරු ආසාදන ඖෂධීය ක්‍රම භාවිතා නොවන මෙම පර්යේෂණයේ දී පවා සංඛ්‍යායනිකව සැලකිය යුතු තරම් වැඩි වේනම් පර්යේෂණය වහාම නවතනු ලැබේ.

මුලදී පර්යේෂණයට සහභාගී වීමට කැමැත්ත ප්‍රකාශ කලද ඔබට අවශ්‍ය විටක හේතු දැක්වීමකින් තොරව පර්යේෂණයෙන් ඉවත් වීමට හැකියි. එසේ නම් ඔබ ඒ බව අප වෙත දැන්වීමට කාරුණික විය යුත්තේ ඔබේ දත්ත පර්යේෂණයෙන් ඉවත් කල යුතු නිසාවෙනි.

මෙම පර්යේෂණයේ දී ලේටෙක්ස් සහිත අත්වැසුම් භාවිත වෙනු ඇත. සමහර අයට ලේටෙක්ස් අසාත්මිකතාවන් පවතියි. සුලු අසාත්මිකතාවන්ට අදාල ප්‍රතිකාර ලබා දෙන මුත් දරුණු අසාත්මිකතවක් ඇති වුවහොත් ඔබට වහාම අදාල ප්‍රතිකාර ලබා දෙන අතර පර්යේෂණයෙන් ඉවත් කරනු ලැබේ.

අධික ස්නායු උත්තේජනයක්, ගැබ් ගෙල තුවාල වීමක්, ගර්භාශය තුවාල වීමක් හෝ විෂබීජ ශරීරගත වීමක් සිදු වුවහොත් ඔබව පර්යේෂණයෙන් ඉවත් කරනු ලැබේ.

ගැබ් ගෙල සූදානම් කිරීමේ ක්‍රමය මුලින් සිදු කිරීමට පෙර ඔබට ප්‍රසව වේදනාව ඇති වුවහොත් ඔබව පර්යේෂණයෙන් ඉවත් කරනු ලැබේ.

යම් හෙයකින් හදිසි ප්‍රසූතියක් අවශ්‍ය වුවහොත් ඒ සඳහා ඔබව පර්යේෂණයෙන් ඉවත් කරනු ලැබේ.

පර්යේෂණයෙන් ඉවත් කලහොත් ඔබව අදාල තත්වයන්ට සාමාන්‍යයෙන් ප්‍රතිකාර කරන ආකාරයටම සියලු ප්‍රතිකාර ලබා දෙනු ලැබේ.

පර්යේෂණයෙන් ඉවත් කලොත් ඔබේ දත්ත පර්යේෂණයේන් ඉවත් කරන අතර ගණනය කිරීම් වලට යොදා ගනු නොලැබේ.

අවශ්‍ය පුද්ගල සංඛ්‍යාව සම්පූර්ණ කිරීම පිණිස වෙනත් අයෙකු ඔබ වෙනුවට පර්යේෂණයට බඳවා ගනු ලැබේ.

1. **ඔබට තොරතුරු ලබා ගත හැකි ක්‍රම**

ඔබට අවශ්‍ය පරිදි අපෙන් තොරතුරු විමසිය හැකියි. හෙද හා වෛද්‍ය කාර්ය මන්ඩලයෙන් ප්‍රශ්න ඇසිය හැකියි. අපේ දුරකථන අංකය මෙහි සඳහන් වේ. අවශ්‍ය නම් රෝහලට පැමිණ අප මුනගැසීමට ද ඔබට හැකියි. **Dr. Sameera -0776748181**

**මෙම පර්යේෂණය කොළඹ විශ්ව විද්‍යාලයේ, වෛද්‍ය පීඨයේ, පර්යේෂණ ආචාර ධර්ම සමාලෝචන කමිටුව මඟින් අනුමත් කර ඇත. ඔබට තවත් තොරතුරු අවශ්‍ය නම් හෝ, පැමිණිළි ඉදිරිපත් කිරීමට අවශ්‍ය නම් දුරකථන අංක 0112695300 දිගුව 240 (පෙ.ව 9 සිට ප.ව 4 දක්වා) හෝ ඊ තැපැල්** [**info.ethics@med**](mailto:info.ethics@med)**.cmb.ac.lk ඇමතිය හැක.**

**Annexure B – Sinhala version of the consent form**

**Membrane sweep සහ Cervical massage මඟින් ගැබ් ගෙල දරු ප්‍රසූතියක් සඳහා සූදානම් කිරීමේ සාර්ථකත්වය සහ මාතෘ තෘප්තිමත්භාවය සසඳා බැලීම**

**කැමැත්ත ප්‍රකාශ කිරීම**

**A කොටස - මව විසින් පිරවිය යුතුයි.**

**මෙම සම්පූර්ණ පත්‍රිකාව ම මව විසින් පිරවිය යුතුය.**

1. ඔබ තොරතුරු පත්‍රිකාව හොඳින් කියෙවුවා ද? (ඔබට පිටපතක් ලබ දෙනු ඇත)

ඔවු/නැහැ

1. මෙම පර්යේෂණය සම්බන්ධ ව සාකච්ඡා කිරීමට හ ප්‍රශ්ණ ඇසීමට අවස්ථාවක් ලැබුනා ද? ඔවු/නැහැ
2. ඔබගේ ප්‍රශ්ණ වලට සතුටුදායක මට්ටමෙන් පිළිතුරු ලැබුනා ද? ඔවු/නැහැ
3. ඔබට පර්යේෂණය සම්බන්ධයෙන් ප්‍රමාණවත් තරම් තොරතුරු ලැබුනා ද? ඔවු/නැහැ
4. ඔබට තොරතුරු පැහැදිලි කලේ කවු ද?....................................................................................
5. ඔබට අවශ්‍ය විටක, හේතු නොදක්වා මෙම පර්යේෂණයෙන් ඉවත් විය හැකි බවත්, එයින් ඔබගේ සෞඛ්‍ය ක්‍රියාවලියට කිසිදු බලපෑමක් ඇති නොවන බවත් ඔබට

පැහැදිලි ද? ඔවු/නැහැ

1. ඔබගේ සහභාගිත්වයට අදාල තොරතුරු පර්යේෂණ සහායකයින් අතට පත් වනු ඇත. ඔබගේ සියලු පෞද්ගලික තොරතුරු අතිශය රහසිගත් ලෙස සැලකෙනු ඇත. ඔබගේ තොරතුරු භාවිතා කිරීමට පර්යේෂකයින්ට අවසර දෙනවා ද? ඔවු/නැහැ
2. ඔබට මෙම තීරණය ගැනීමට ප්‍රමාණවත් තරම් කාලයක් ලැබුනා ද? ඔවු/නැහැ
3. ඔබ මෙම පර්යේෂණයට සහභාගි වීමට කැමැත්ත ප්‍රකාශ කරනවා ද? ඔවු/නැහැ

මවගේ අත්සන………………………………………………… දිනය………………………………………….

නම……………………………………………………………………………………………………………………………………………………

**B කොටස - මෙම කොටස පර්යේෂකයින් විසින් පිරවිය යුතුයි.**

ඉහත නම සඳහන් මව වෙත මා විසින් පර්යේෂණය ගැන පැහැදිලි කර දෙනු ලැබූ අතර ඇය පර්යේෂණයට සහභාගි වීමට කැමැත්ත ප්‍රකාශ කරන ලදී.

පර්යේෂකයාගේ අත්සන………………………………………….. දිනය…………………………………………………

නම……………………………………………………………………………………………………………………………………………

Annexure C – English version of the information sheet

**Comparative effectiveness and maternal acceptability with cervical ripening using membrane sweep and cervical massage: A randomized controlled clinical trial**

**INFORMATION SHEET**

I am Dr. T.M.S.S.B.Madugalle working as a Registrar in Obstetrics and Gynaecology in De Soysa Hospital for Women, Colombo. Currently I am conducting a study comparing two physical methods of preparing the cervix for a vaginal delivery with regard to their effectiveness and maternal acceptability and I cordially invite you to participate in the study. Study activities will occur during your admission at the ward after you admit for delivery of your baby.

1. **Aims**

The main aim of this study is to compare two mechanical methods of preparing the uterine cervix for a vaginal delivery. We will be comparing,

- 1. Number that goes into labour spontaneously
  2. Number that need formal induction of labour
  3. Induction to delivery time
  4. Vaginal delivery rates by 40^th^ and 41^st^ week of pregnancy
  5. Ceasarian section rates
  6. Number that need Oxytocin during labour
  7. Number that gets excessive uterine contractions
  8. Damage to uterus
  9. Maternal fever
  10. Duration of hospital stay
  11. Condition of the baby
  12. Maternal acceptability with regards to each methods of induction.

1. **Participation of voluntary**

You may withdraw previously given consent and withdraw from the study at any moment during the study period. In case of such voluntary withdrawal, please inform the study team immediately after the decision to withdraw has been made. This is to ensure that your data obtained will be destroyed and not included in statistical analysis.

1. **Duration, procedures of the study and participant’s responsibilities**

We will divide selected mothers into three groups randomly. First group will get membrane sweep, second group will get cervical massage and the third group is the control group without any intervention. Cervical massage means stimulating the uterine cervix for 15-30 seconds during a vaginal examination. Membrane sweep means separating the membranes from the lower uterine wall by running the examining finger between the membranes and the uterine wall three times during a vaginal examination. Both these methods may cause a mild pain and a mild vaginal bleeding. We will perform the selected procedure at 39^th^ week and in one week if spontaneous labour doesn’t follow. We will follow you up till discharge after delivery. We will assess the status of your cervix when you have 2 contractions of moderate strength. If your cervix is unfavorable you will be managed according to guidelines and reassessed as required. If your cervix is favorable, you will be taken into the labor ward and we will let labor progress. If you do not go into labour by 40+6 weeks you will be reassessed. Ward obstetrician will decide on formal induction. You will be followed up till discharge after delivery.

1. **Potential benefits**

Formal induction of labour with mechanical and pharmacological agents carry a significant risk of uterine hyperstimulation, fetal distress, uterine rupture and operative deliveries. Cervical ripening has been shown to reduce the need for formal induction thereby reducing above risks. By participating in the study, you will have the clear direct benefit of not facing risks of formal induction.

1. **Risks, hazards and discomforts**
   1. Risks include allergies to latex material of examination gloves, episodes of low heart rate and blood pressure, damage to your uterus and infections.
   2. All the procedure, including the investigated procedures are a part of routine obstetric practice. Therefore, the study participants will not be placed under additional risk for the sake of the study.
   3. If you get an allergic reaction to latex you will treated for it, kept in ward and monitored for 24 hours. If the allergic reaction is severe you will be withdrawn from the study.
   4. If you get an episode of low heart ate and blood pressure, no further procedures will be performed on you. You will be taken off the study and treated accordingly.
   5. If your cervix gets damaged, we will immediately repair it.
   6. If your uterus gets damaged, immediate surgery will be performed. We may be able to repair it or rarely uterus may be removed if absolutely necessary.
   7. If you catch an infection you will be given antibiotics. We will get other specialists involved in your care.
   8. If any psychological concerns arise relevant referrals will be made and a care plan will be agreed upon.
   9. In case of any concern participants will be advised to visit unit clinics for follow up care after the study.
   10. In case of any adverse event mentioned above or unexpected adverse event occurs, the Ethical review committee and all the participants will be informed immediately. If requested by the ERC a re-consenting will be conducted.
2. **Compensation, re-imbursement, incentives for study participants**

Participation will be purely voluntary with full understanding of the minimal risks associated with mechanical induction of labour and there will be no incentives for participation or financial compensation for any injury related to the conduct of the study.

1. **Confidentiality**

The data sheets will be handled only by the investigators and will be kept under lock and key while not in use. During publication, identity of participants will not be revealed. Following completion of the trial anonymized study data and the study protocol will be made available publicly under [CC BY 4.0](https://creativecommons.org/licenses/by/4.0/) license on an established research data repository. Medical records (Bed Head Ticket) will be stored at the hospital as per routine regulations and the investigators will only keep anonymized study data.

1. **Termination of the study and withdrawal of participants**

The study will be terminated if these mechanical methods of inductions would impose unreasonable and statistically significant risk. These risks include uterine rupture, cervical injuries, maternal infections and neonatal injury. If these complications occur at a statistically significant level the study will be terminated.

You may withdraw previously given consent and withdraw from the study at any moment during the study period. In case of such voluntary withdrawal, please inform the study team immediately after the decision to withdraw has been made. This is to ensure that the data obtained will be destroyed and not included in statistical analysis.

You will be withdrawn from the study if severe Latex allergies, vasovagal attacks, cervical injuries, uterine rupture or maternal infections occur.

You will be removed from the study if they go into active labour (Achieve a Modified Bishops’ Score 7≤) before the induction in undertaken.

You will be withdrawn from the study if they develop an indication for immediate delivery before the induction is undertaken.

If withdrawn you will be managed according to the routine ward protocol.

If you withdraw from the study, your data sheet will be destroyed and will not be used in the analysis.

To complete the sample size requirements, further participants will be recruited to replace you.

1. **Clarifications**

You can contact us at any time. We have mentioned our contact details here. Feel free to visit us at the hospital if you prefer.

Dr. Sameera – 0776748181

**This project has been approved by the Ethics Review Committee, Faculty of Medicine, University of Colombo. You may contact the committee if you wish to seek clarifications, record any concerns or make complaints about the study by calling 0112695300 extension 240 (between 9am and 4pm) or by sending an email to** [**info.ethics@med.cmb.ac.lk**](mailto:info.ethics@med.cmb.ac.lk)

**Annexure D – English version of the consent form**

**Comparative effectiveness and maternal acceptability with cervical ripening using membrane sweep and cervical massage: A randomized controlled clinical trial**

**CONSENT FORM**

**Part A – To be filled by the participant**

The participant should complete the whole of this sheet herself.

1. Have you read the information sheet? (Please keep a copy for yourself) YES/NO
2. Have you had an opportunity to discuss this study and ask any questions? YES/NO
3. Have you had satisfactory answers to all your questions? YES/NO
4. Have you received enough information about the study? YES/NO
5. Who explained the study to you?

………………………………………………………………..

1. Do you understand that you are free to withdraw from the study at any time, without having to give a reason and without affecting your future medical care? YES/NO
2. Information held by the investigators relating to your participation in this study may be examined by other research assistants. All personal details will be treated as STRICTLY CONFIDENTIAL. Do you give your permission for these individuals to have access to your records? YES/NO
3. Have you had sufficient time to come to your decision? YES/NO
4. Do you agree to take part in this study? YES/NO

Participant’s signature: ………………………….. Date:………………………………….

Name (BLOCK CAPITALS)

…………………………………………………………………………………

**Part B – To be filled by the investigator**

I have explained the study to the above volunteer and she has indicated her willingness to take part.

Signature of investigator: ……………………………… Date: ………………………………

Name (BLOCK CAPITALS):

…………………………………………………………………………………………………

**Annexure E – Tamil version of information sheet**

**மெம்பரன் ஸ்வீப் மற்றும் கர்ப்பப்பை வாய் மசாஜ் பயன்படுத்தி கர்ப்பப்பை வாய்ந்த பழுப்பு நிறத்துடன் ஒப்பிடுவதன் விளைவாக மற்றும் தாய் திருப்தி: ஒரு சீரற்ற கட்டுப்படுத்தப்பட்ட மருத்துவ சோதனை**

**தகவல் தாள்**

நான் டாக்டர். டி.எஸ். எஸ்.எஸ்.பீ.மடுகல்லே, கொழும்பிலுள்ள மகளிர் டி சாய்சா மருத்துவமனையில் உள்ள மகப்பேறியல் மற்றும் பெண்ணோயியல் உள்ள பதிவாளராக பணிபுரிகிறார். தற்போது நான் அவர்களின் இயல்பான மற்றும் தாய்வழி திருப்தி தொடர்பாக ஒரு யோனி டெலிவரிக்கு கருப்பை வாய் தயார் இரண்டு உடல் முறைகள் ஒப்பிடுகையில் ஒரு ஆய்வு நடத்தி நான் இதயப்பூர்வமாக ஆய்வு பங்கேற்க அழைக்கிறேன். உங்கள் குழந்தை வழங்குவதற்கு ஒப்புக் கொண்டபிறகு, வார்டுகளில் உங்கள் சேர்க்கை நடக்கும் போது படிக்கும் நடவடிக்கைகள் நடக்கும்.

1. **நோக்கங்கள்**

இந்த கருத்தின் முக்கிய குறிக்கோள், கருப்பை வாய் வழியாக ஒரு யோனி டெலிவரிக்கு தயாரிக்கும் இரண்டு இயந்திர முறைகள் ஒப்பிடுவதாகும். நாம் ஒப்பிட்டு,

1. யோனி பிறப்புக்கு தன்னிச்சையாக செல்லும் எண்
2. யோனி பிறப்புக்கு தன்னிச்சையாக செல்லாத எண்ணிக்கை
3. செயல்முறை முதல் யோனி பிறப்பு வரை நேரம்
4. 40 வாரங்கள் மற்றும் 41 வாரங்களில் யோனி பிரசவத்தின் சதவீதம்
5. சீசர் பிரிவு விகிதம்
6. ஆக்ஸிடாஸின் அதிகரிப்பு விகிதம்
7. கருப்பை உயர் இரத்த அழுத்தம்
8. கருப்பை முறிவு
9. தாய்வழி காய்ச்சல்
10. மருத்துவமனையின் காலம் நீடிக்கும்
11. குழந்தையின் நிலை
12. தூண்டுதலின் ஒவ்வொரு முறைகள் தொடர்பாக தாய் திருப்தி
13. **தன்னார்வ பங்கேற்பு**

ஆய்வின் காலத்தில் எந்தவொரு நேரத்திலும் நீங்கள் பெற்ற ஒப்புதல் மற்றும் பின்விளைவுகளை திரும்ப பெறலாம். அத்தகைய தன்னார்வத் தொகை திரும்பப் பெறப்பட்டால், திரும்பப் பெற முடிந்தவுடன் உடனடியாக ஆய்வுக் குழுவை தயவுசெய்து தெரிவிக்கவும். பெறப்பட்ட உங்கள் தரவு அழிக்கப்படும் மற்றும் புள்ளிவிவர பகுப்பாய்வு சேர்க்கப்படவில்லை என்பதை உறுதி செய்ய வேண்டும்.

1. **காலம், ஆய்வு மற்றும் பங்கேற்பாளரின் பொறுப்புகளின் நடைமுறைகள்**
2. தேர்ந்தெடுக்கப்பட்ட தாய்மார்களை தோராயமாக மூன்று குழுக்களாகப் பிரிப்போம். முதல் குழு பெறுகிறது Membrane sweeping, இரண்டாவது குழு பெறுகிறது cervical massage மற்றும் மூன்றாவது குழுவிற்கு எந்த தலையீடும் கிடைக்காது. கர்ப்பப்பை வாய் மசாஜ் ஒரு கருப்பை பரிசோதனை போது 15-30 விநாடிகள் கருப்பை கருப்பை தூண்டுதல் பொருள். மெம்பிரேன் ஸ்வீப் என்பது சர்க்கரை சுவரில் இருந்து சவ்வுகளை பிரிப்பதன் மூலம், சவ்வு மற்றும் கருப்பை சுவருக்கு இடையே மூன்று முறை யோனி பரிசோதனைக்கு இடையில் பரிசோதனையின் விரலை இயக்கும். இந்த இரண்டு நடைமுறைகளும் லேசான வலி மற்றும் லேசான யோனி இரத்தப்போக்கு ஏற்படலாம். அனைத்து கர்ப்பிணித் தாய்மார்களிலும், தாய்க்கு பிரசவம் இல்லை என்றால் 39 வாரங்களிலும் ஒரு வாரத்திலும் செயல்முறை செய்கிறோம். பிரசவத்திற்குப் பின் உங்களை வெளியேற்றும் வரை பின்தொடர்வோம். பிரசவ வலி, 10 நிமிட இடைவெளியில் இரண்டு மிதமான கருப்பைச் சுருக்கங்கள் இருக்கும்போது, ​​கர்ப்பத்தின் சாத்தியத்தை மதிப்பிடுகிறோம். யோனி பிறப்புக்கு நீங்கள் இன்னும் பொருத்தமானவர் இல்லை என்று நாங்கள் கண்டால், பின்னர் நாங்கள் உங்களை ஆராய்வோம். கர்ப்பப்பை வாய்ந்த நிலை திருப்திகரமாக இருந்தால், உழைப்பு அறையில் உங்களை ஏற்றுக்கொள்வோம், மற்றும் யோனி டெலிவரிக்கு யூனிட் நெறிமுறைகளின்படி உங்களை நிர்வகிக்கலாம். நீங்கள் 40 6 வாரங்களுக்குள் பிரசவத்திற்குச் செல்லவில்லை என்றால் நீங்கள் மறு மதிப்பீடு செய்யப்படுவீர்கள். வார்டில் உள்ள பெரும்பாலான மூத்த மருத்துவர்கள் பின்னர் என்ன செய்வது என்று முடிவு செய்வார்கள். பிரசவத்திற்குப் பிறகு வெளியேற்றப்படும் வரை நீங்கள் பின்தொடரப்படுவீர்கள்.
3. **சாத்தியமான நன்மைகள்**

இயந்திர மற்றும் மருந்தியல் முகவர்களுடன் உழைப்பின் முறையான தூண்டுதல் கருப்பை ஹைப்பர்ஸ்டிமுலேஷன், கருவின் துன்பம், கருப்பை சிதைவு மற்றும் செயல்பாட்டு பிரசவங்களுக்கு குறிப்பிடத்தக்க ஆபத்தை ஏற்படுத்துகிறது. கர்ப்பப்பை வாய் பழுக்க வைப்பது முறையான தூண்டுதலின் தேவையைக் குறைப்பதாகக் காட்டப்பட்டுள்ளது, இதன் மூலம் மேலே உள்ள அபாயங்களைக் குறைக்கிறது. ஆய்வில் பங்கேற்பதன் மூலம், முறையான தூண்டலின் அபாயங்களை எதிர்கொள்ளாததன் தெளிவான நேரடி நன்மை உங்களுக்கு கிடைக்கும்.

1. **அபாயங்கள், அபாயங்கள் மற்றும் அபாயங்கள்**
   1. அபாயங்கள் மரப்பால் ஒவ்வாமை, குறைந்த இதய துடிப்பு மற்றும் இரத்த அழுத்தத்தின் அத்தியாயங்கள், உங்கள் கருப்பையில் சேதம் மற்றும் தொற்றுநோய்கள்.
   2. விசாரிக்கப்பட்ட நடைமுறைகள் உட்பட அனைத்து நடைமுறைகளும் வழக்கமான மகப்பேறியல் பயிற்சியின் ஒரு பகுதியாகும். எனவே, ஆய்வில் பங்கேற்பாளர்கள் ஆய்வின் பொருட்டு கூடுதல் ஆபத்தில் வைக்கப்பட மாட்டார்கள்.
   3. லேடெக்ஸிற்கு உங்களுக்கு ஒவ்வாமை ஏற்பட்டால், அதற்கு நீங்கள் சிகிச்சை அளிப்பீர்கள், வார்டில் வைத்து 24 மணி நேரம் கண்காணிக்கப்படுவீர்கள். ஒவ்வாமை எதிர்வினை கடுமையாக இருந்தால் நீங்கள் ஆய்வில் இருந்து விலக்கப்படுவீர்கள்.
   4. குறைந்த இதய துடிப்பு மற்றும் இரத்த அழுத்தத்தின் ஒரு அத்தியாயத்தைப் பெற்றால், மேலதிக நடைமுறைகள் உங்களிடம் செய்யப்படாது. நீங்கள் படிப்பிலிருந்து வெளியேற்றப்பட்டு அதற்கேற்ப சிகிச்சை பெறுவீர்கள்.
   5. உங்கள் கருப்பை வாய் சேதமடைந்தால், நாங்கள் அதை உடனடியாக சரிசெய்வோம்.
   6. உங்கள் கருப்பை சேதமடைந்தால், உடனடியாக அறுவை சிகிச்சை செய்யப்படும். நாம் அதை சரிசெய்ய முடியும் அல்லது முற்றிலும் தேவைப்பட்டால் அரிதாக கருப்பை அகற்றப்படலாம்.
   7. உங்களுக்கு தொற்று ஏற்பட்டால் உங்களுக்கு நுண்ணுயிர் எதிர்ப்பிகள் வழங்கப்படும். உங்கள் பராமரிப்பில் மற்ற நிபுணர்களை நாங்கள் ஈடுபடுத்துவோம்.
   8. எந்தவொரு உளவியல் சம்பந்தமும் எழுந்தால், உரிய பரிந்துரைகளை மேற்கொள்ள வேண்டும் மற்றும் ஒரு பராமரிப்பு திட்டம் ஏற்றுக்கொள்ளப்படும்.
   9. எந்தவொரு கவலையும் ஏற்பட்டால், படிப்பிற்குப் பிறகு கவனத்தை கவனிப்பதற்காக அலகு கிளினிக்குகள் வருவதற்கு அறிவுறுத்தப்படுவார்கள்.
   10. மேலே குறிப்பிட்டுள்ள தீங்கு விளைவிக்கும் நிகழ்வு அல்லது சந்தேகத்திற்கு இடமில்லாத பாதகமான நிகழ்வு ஏற்படுகையில், தார்மீக மறு ஆய்வு குழு மற்றும் அனைத்து பங்கேற்பாளர்களும் உடனடியாக தெரிவிக்கப்படுவார்கள். ERC கோரினால் மீண்டும் ஒப்புதல் அளிக்கப்படும்
2. **ஆய்வு, மறுநிதி, ஆய்வு பங்கேற்பாளர்களுக்கு ஊக்குவிப்பு**

உழைப்பு இயந்திர தூண்டுதலுடன் தொடர்புடைய குறைந்தபட்ச அபாயங்களை முழுமையாக புரிந்துகொள்வதன் மூலம் பங்கேற்பு முற்றிலும் தன்னார்வமாக இருக்கும். ஆய்வின் நடத்தை தொடர்பான எந்தவொரு காயத்திற்கும் பங்கேற்பு அல்லது நிதி இழப்பீட்டுக்கு எந்த ஊக்கமும் இல்லை.

1. **ரகசியத்தன்மை**

தரவுத் தாள்கள் மட்டுமே விசாரணைக்கு உட்படுத்தப்படும் மற்றும் பயன்பாட்டில் இல்லாதபோது பூட்டு மற்றும் விசை ஆகியவற்றின் கீழ் வைக்கப்படும். வெளியீட்டின் போது, ​​பங்கேற்பாளர்களின் அடையாளம் வெளிப்படுத்தப்படாது. விசாரணையின் முடிவைத் தொடர்ந்து, விசாரணைகள் மற்றும் ஒழுங்குமுறை அதிகாரிகள் வெளியீடுகளுக்கு வெளியே தரவு மற்றும் கண்டுபிடிப்புகள் ஆகியவற்றைப் படிக்க அணுக முடியும். மருத்துவ பதிவேடுகள் (பெட் ஹெட் டிக்கெட்) வழக்கமான விதிமுறைகளின்படி மருத்துவமனையில் சேமிக்கப்படும் மற்றும் புலன்விசாரணை மட்டும் ஆய்வு தரவு வைத்திருக்கும். அனைத்து தரவு மற்றும் ஆய்வு பொருள் விசாரணை முன்னிலையில் தொடர்புடைய அதிகாரம் முறையான கோரிக்கை மீது கிடைக்கும். படிப்புத் தகவல்கள் 3 வருட காலத்திற்கு சேமித்து வைக்கப்படும், பின்னர் துண்டாக்கப்பட்டன மற்றும் அழிக்கப்படும்.

1. **ஆய்வு முடித்தல் மற்றும் பங்கேற்பாளர்கள் திரும்பப் பெறுதல்**

தூண்டுதல் இந்த இயந்திர முறைகளை நியாயமற்ற மற்றும் புள்ளிவிவர குறிப்பிடத்தக்க அபாயத்தை சுமத்தினால் இந்த ஆய்வு நிறுத்தப்படும். இந்த அபாயங்கள் கருப்பைச் சிதறல், கர்ப்பப்பை வாய் காயங்கள், தாய் நோய்த்தாக்கம் மற்றும் குழந்தை பிறந்த காயம் ஆகியவை அடங்கும். இந்த சிக்கல்கள் ஒரு புள்ளியியல் குறிப்பிடத்தக்க மட்டத்தில் நடந்தால், ஆய்வு நிறுத்தப்படும்.

ஆய்வின் காலத்தில் எந்தவொரு நேரத்திலும் நீங்கள் பெற்ற ஒப்புதல் மற்றும் பின்விளைவுகளை திரும்ப பெறலாம். அத்தகைய தன்னார்வத் தொகை திரும்பப் பெறப்பட்டால், திரும்பப் பெற முடிந்தவுடன் உடனடியாக ஆய்வுக் குழுவை தயவுசெய்து தெரிவிக்கவும். பெறப்பட்ட தரவு அழிக்கப்பட்டு, புள்ளிவிவர பகுப்பாய்வுக்குள் சேர்க்கப்படவில்லை என்பதை உறுதி செய்ய வேண்டும்.

கடுமையான லேடெக்ஸ் ஒவ்வாமை, வாசோவாகல் தாக்குதல்கள், கர்ப்பப்பை வாய்ப் காயங்கள், கருப்பைச் சிதைவு அல்லது தாய் நோய்த்தொற்றுகள் ஆகியவற்றால் நீங்கள் ஆய்வுக்கு உட்படுத்தப்படுவீர்கள்.

நீங்கள் செயலில் உள்ள உழைப்புக்கு சென்றால் (நீங்கள் ஒரு திருத்தப்பட்ட ஆயர்கள் ‘ஸ்கோர் 7 புள்ளிகளை அடைய வேண்டும்) மேற்கொள்வதற்கு முன், நீங்கள் படிப்பிலிருந்து அகற்றப்படுவீர்கள்.

தூண்டுதலுக்கு முன்னர் உடனடியாக வழங்குவதற்கான ஒரு அறிகுறியை அவர்கள் வளர்த்துக் கொண்டால், நீங்கள் ஆய்வுக்குப் பின் திரும்பப் பெறுவீர்கள்.

திரும்பப் பெறப்பட்டால், வழக்கமான வார்டு நெறிமுறையின்படி நீங்கள் நிர்வகிக்கப்படுவீர்கள்.

ஆய்வில் இருந்து நீங்கினால், உங்கள் தரவு தாள் அழிக்கப்பட்டு, பகுப்பாய்வில் பயன்படுத்தப்படாது.

மாதிரி அளவு தேவைகளை பூர்த்தி செய்ய, நீங்கள் மாற்றுவதற்கு கூடுதல் பங்கேற்பாளர்கள் நியமிக்கப்படுவார்கள்.

1. **தெளிவுரைகள்**

நீங்கள் எந்த நேரத்திலும் எங்களை தொடர்பு கொள்ளலாம். இங்கே எங்கள் தொடர்பு விவரங்களை குறிப்பிட்டுள்ளோம். நீங்கள் விரும்பினால் மருத்துவமனையில் எங்களை பார்க்க தயங்க.

டாக்டர் சமீரா – 0776748181

**இந்த திட்டம் கொழும்பின் பல்கலைக்கழகத்தின் மருத்துவ பீடத்தின் நெறிமுறை மறு ஆய்வு குழுவால் அங்கீகரிக்கப்பட்டுள்ளது. 0112695300 நீட்டிப்பு 240 (காலை 9 மணி முதல் மாலை 4 மணி வரை) அல்லது** [**info.ethics@med**](mailto:info.ethics@med)**.cmb.ac.lk க்கு மின்னஞ்சலை அனுப்புவதன் மூலம் நீங்கள் விளக்கங்களைத் தேட வேண்டுமெனில்,**

**Annexure F– Tamil version of consent form**

**மெம்பரன் ஸ்வீப் மற்றும் கர்ப்பப்பை வாய் மசாஜ் பயன்படுத்தி கர்ப்பப்பை வாய்ந்த பழுப்பு நிறத்துடன் ஒப்பிடுவதன் விளைவாக மற்றும் தாய் திருப்தி: ஒரு சீரற்ற கட்டுப்படுத்தப்பட்ட மருத்துவ சோதனை**

**ஒப்புமை படிவம்**

**பகுதி A – பங்கேற்பாளரால் நிரப்பப்பட வேண்டும்**

பங்குதாரர் இந்த தாளின் முழுவதையும் முடிக்க வேண்டும்.

1. தகவல் தாள் நீங்கள் வாசித்திருக்கிறீர்களா? (தயவுசெய்து ஒரு நகலை வைத்திருங்கள்) ஆம் / இல்லை
2. இந்த ஆய்வில் கலந்துரையாட மற்றும் ஏதேனும் கேள்விகள் கேட்க உங்களுக்கு வாய்ப்பு கிடைத்ததா? ஆம் /இல்லை
3. உங்கள் எல்லா கேள்விகளுக்கும் நீங்கள் திருப்திகரமான பதில்களைக் கொண்டிருந்தீர்களா? ஆம் /இல்லை
4. படிப்பிற்கான போதுமான தகவலை நீங்கள் பெற்றுள்ளீர்களா?

ஆம் /இல்லை

1. உங்களைப் பற்றி யார் விளக்கினார்?........................................................................ ..
2. எப்போது வேண்டுமானாலும் படிப்பில் இருந்து விலகி விடுவதால், உங்கள் எதிர்கால மருத்துவ சிகிச்சை பாதிக்கப்படாமல், ஒரு காரணத்தைத் தெரிவிக்காமலும், ஆம் /இல்லை
3. இந்த ஆய்வில் உங்கள் பங்கு தொடர்பான விசாரணையாளர்கள் நடத்திய தகவல் பிற ஆராய்ச்சி உதவியாளர்களால் ஆராயப்படலாம். அனைத்து தனிப்பட்ட விவரங்களையும் கண்டிப்பாக ரகசியமாக கருதலாம். உங்கள் பதிவுகளை அணுகுவதற்கு இந்த நபர்களுக்கு உங்கள் அனுமதியைக் கொடுக்கிறீர்களா? ஆம் /இல்லை
4. உங்கள் தீர்மானத்திற்கு வருவதற்கு உங்களுக்கு போதுமான நேரம் இருந்ததா? ஆம் /இல்லை
5. இந்த ஆய்வில் கலந்துகொள்ள நீங்கள் ஒப்புக்கொள்கிறீர்களா?

ஆம் /இல்லை

பங்கேற்பாளரின் கையொப்பம்: …........................... .. தேதி: …………….....................................

பெயர் (BLOCK CAPITALS) ……………………………...........................................................................................

**பகுதி B – புலன்விசாரணை மூலம் நிரப்பப்பட வேண்டும்**

மேலே உள்ள தன்னார்வலரிடம் ஆய்வுகளை விளக்கினேன், அவள் பங்கேற்க விருப்பம் தெரிவித்தார்.

புலன்விசாரணை கையெழுத்து: ….............................. … தேதி: ….................................

பெயர் (BLOCK CAPITALS):…............................................................................................................

Annexure G – Sinhala version of data collection form

1. A කොටස - ජීව දත්ත
   1. වයස:……………………………………………………………………………….
   2. කීවෙන වරට ගැබ් ගත්තේ ද යන වග:……………………………………………….
   3. ගැබට සති 28 පසු වෙන තෙක් පැවති කෙවෙනි ගර්භණී බවද යන වග:………………
   4. වර්තමාන ගැබට සති ගණන:……………………….උස:………… බර:………….
   5. BMI අගය:…………………..
   6. පෙර ගර්භණීභාවයන්, උපත් බර, දරුවා ලැබුනු ක්‍රමය, හේතුව

| # | දරුවා ලැබුනු ක්‍රමය | උපත් බර | හේතුව |
| --- | --- | --- | --- |
|  |  |  |  |
|  |  |  |  |
|  |  |  |  |
|  |  |  |  |
|  |  |  |  |

1. B කොටස – ගැබ් ගෙල සූදානම් කිරීමේ ක්‍රියාවලියේ ප්‍රගතිය

| සති 39 / 40 දී මූලික Modified Bishops’ Score | සති 40 දී දෙවැනි Modified Bishops’ Score | පටිපාටිය සිදු කල දින හා වෙලාව | සති 40+6 දී Modified Bishops’ Score |
| --- | --- | --- | --- |
|  |  |  |  |

1. C කොටස - උපත් විස්තර
   1. දරුවා ලැබුනු ක්‍රමය:………………………………………………………………
   2. දරුවා ලැබුනු වෙලාව:………………………………………………………………
   3. Oxytocin මගින් උපත් ක්‍රියාවලිය ඉක්මන් කිරීම:……………………………….
   4. උපරිම ගර්භාෂ සංකෝචන සංඛ්‍යාතය:…………………………………………
   5. ගනණය කරන ලද රැධිර වහනය………………………………………………….
   6. විනාඩි 5 දී APGAR අගය:………………………………………………
2. D කොටස - මවගේ තෘප්තිමත් බව
   1. Membrane sweep හා Cervical massage පිළිබඳව ඔබට පැහැදිලි කර දුන් පසු ඔබට ඒ ගැන බියක් හෝ අවිනිශ්චිතතාවක් දැනුනාද?
      1. දැඩි ලෙස එකඟයි O
      2. එකඟ නොවෙමි O
      3. පැහැදිලි අදහසක් නැත / සිතා බලා නොමැත O
      4. එකඟ වෙමි O
      5. දැඩි සේ විරුද්ධ වෙමි O
   2. එම ගැබ් ගෙල සූදානම් කිරීමේ ක්‍රමය සිදුකරන විට ඔබට දැනුනු අපහසුතාව/ වේදනාව පහත් Wong Baker Pain scale මත ලකුණු කරන්න.


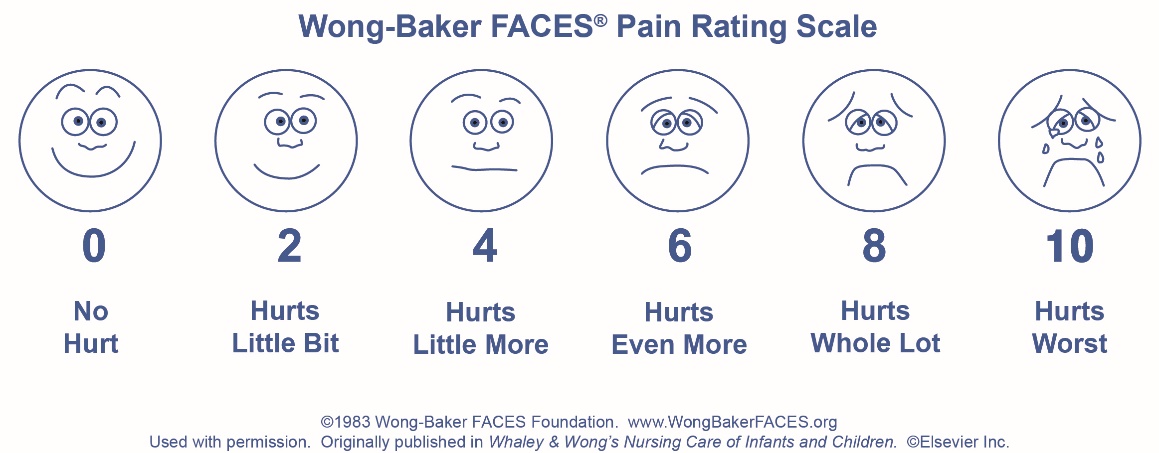


- 1. ඔබට සිතෙන ආකාරයට එම ගැබ්ගෙල සූදානම් කිරීමේ ක්‍රමය සාර්ථක ද?
     1. දැඩි ලෙස එකඟයි O
     2. එකඟ නොවෙමි O
     3. පැහැදිලි අදහසක් නැත / සිතා බලා නොමැත O
     4. එකඟ වෙමි O
     5. දැඩි සේ විරුද්ධ වෙමි O
  2. ඔබට නැවත වතාවක් මෙම ක්‍රියාමාර්ගය අත්විඳිය යුතු වුවහොත් නැවත් වතාවක් මේ ක්‍රමය අනුගමනය කරනවා ද??
     1. දැඩි ලෙස එකඟයි O
     2. එකඟ නොවෙමි O
     3. පැහැදිලි අදහසක් නැත / සිතා බලා නොමැත O
     4. එකඟ වෙමි O
     5. දැඩි සේ විරුද්ධ වෙමි O

Annexure H – English version of Data collection sheet

1. Section A - General details
   1. Age:…………………………………………………………………………………
   2. Gravidity:…………. Parity:………… Period of amenorrhea:…………………….
   3. Maternal height:…………Weight:………….Body mass index:…………………..
   4. Past pregnancies, Past modes of delivery, Indication for induction

| No | Mode of delivery | Birth weight | Indication |
| --- | --- | --- | --- |
|  |  |  |  |
|  |  |  |  |
|  |  |  |  |
|  |  |  |  |
|  |  |  |  |

1. Section B – Progress of induction

| Initial Modified Bishops’ Score at 39^th^ week / 40^th^ week | Second Modified Bishops’ Score at 40^th^ week | Time & date of cervical ripening procedure | Modified Bishops Score at 40+6 weeks |
| --- | --- | --- | --- |
|  |  |  |  |

1. Section C – Delivery details
   1. Mode of delivery:…………………………………………………………………...
   2. Time and date of delivery:…………………………………………………………
   3. Oxytocin augmentation:…………………………………………………………….
   4. Maximum contraction rate:…………………………………………………………
   5. Estimated blood loss:……………………………………………………………….
   6. APGAR score at 5 minutes:…………………………………………………………
2. Section D – Maternal acceptability
   1. Did you feel scared/ concerned when the intervention was explained to you?
      1. Strongly disagree O
      2. Disagree O
      3. Not sure O
      4. Agree O
      5. Strongly agree O
   2. How would you describe the discomfort during intervention on the following Wong Baker pain scale?


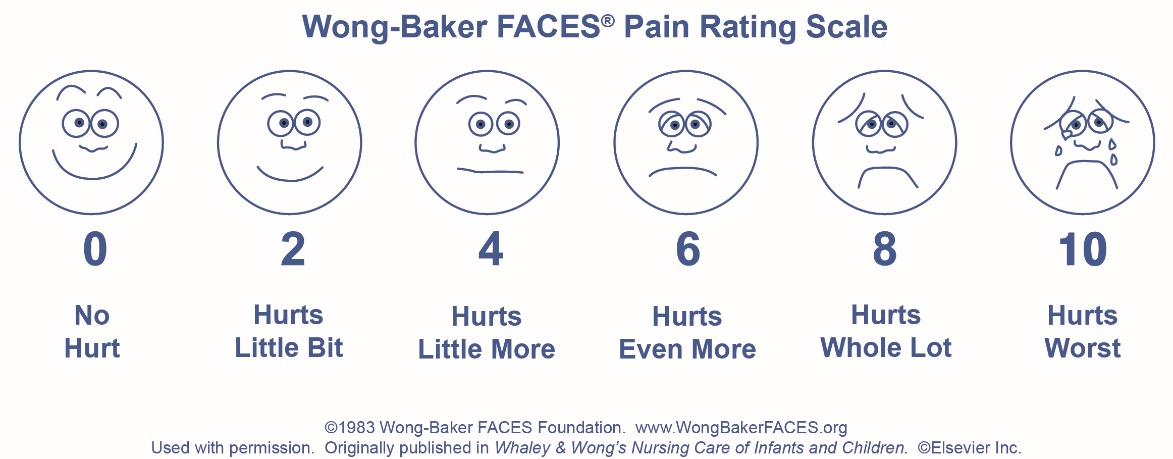


- 1. Do you think the intervention is effective in preparing your cervix for delivery?
     1. Strongly agree O
     2. Agree O
     3. Not sure O
     4. Disagree O
     5. Strongly disagree O
  2. If you were to undergo the same process of induction, would you do it again?
     1. Strongly disagree O
     2. Disagree O
     3. Not sure O
     4. Agree O
     5. Strongly agree O

Annexure I – Tamil version of the data collection form

1. பிரிவு A - உயிர் தரவு
   1. வயது:…………………………………………………………………………….
   2. G:………………………………, P:……………………, POA:……………………
   3. உயரம்:………………………, எடை:…………….., BMI:……………………
   4. முந்தைய கருவுற்றிருக்கும், குழந்தை பிறப்பு வகை,

| # | குழந்தை பிறப்பு வகை | பிறப்பு எடை | காரணம் |
| --- | --- | --- | --- |
|  |  |  |  |
|  |  |  |  |
|  |  |  |  |
|  |  |  |  |
|  |  |  |  |

1. பிரிவு B – முன்னேற்றம்

| 39 மற்றும் 40 வது வாரம் முதல் Modified Bishops’ Score | 40 வது வாரம் இரண்டாவது Modified Bishops’ Score | செயல்முறை நேரம் மற்றும் தேதி | 40 + 6 வாரம் Modified Bishops’ Score |
| --- | --- | --- | --- |
|  |  |  |  |

1. பிரிவு C - பிரசவம் பற்றிய விவரங்கள்
   1. பிரசவம் வகை:…………………………………………………………………………………………
   2. பிறந்த நேரம்:……………………………………………………………………………………………
   3. Oxytocin பெருக்குதல்:……………………………………………………………………………….
   4. அதிகபட்ச கருப்பை சுருக்க விகிதம்:………………………………………………..
   5. மதிப்பிடப்பட்டுள்ளது இரத்த இழப்பு:………………………………………………..
   6. APGAR ஸ்கோர் 5 நிமிடங்களில்:………………………………………………………….
2. பிரிவு D - தாய் திருப்தி
   1. செயல்முறை உங்களுக்கு விளக்கப்பட்டபோது நீங்கள் பயந்தீர்களா?
      1. முரண்படுகிறோம் O
      2. ஒத்திராதே O
      3. ஐயத்திற்கிடமான O
      4. ஒப்புக்கொள் O
      5. வலுவாக ஒப்புக்கொள்கிறேன் O
   2. நடைமுறையின் போது நீங்கள் உணர்ந்த வலியை எவ்வாறு விவரிப்பீர்கள்? பின்வரும் அளவைப் பயன்படுத்தவும்.


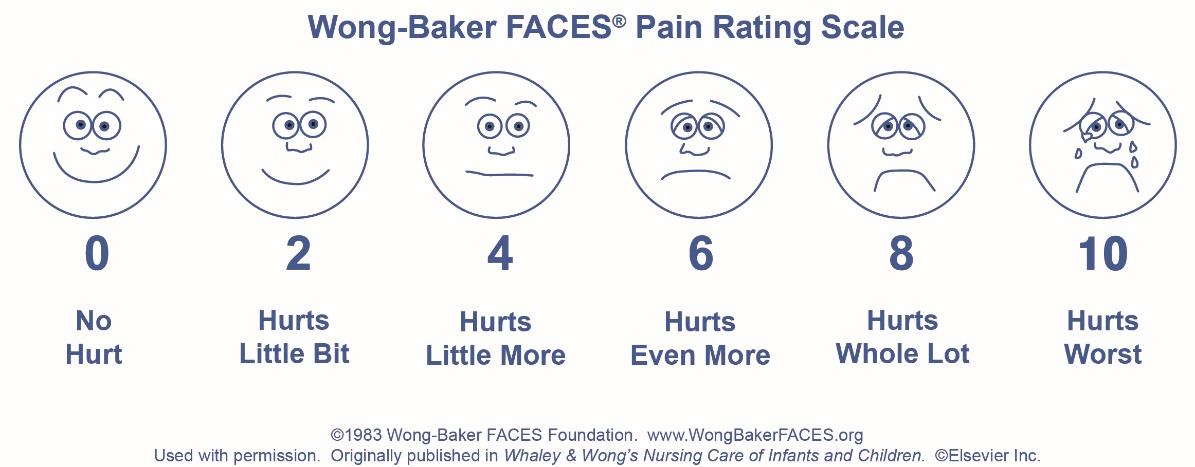


- 1. செயல்முறை பயனுள்ளதாக இருக்கும் என்று நினைக்கிறீர்களா?
     1. முரண்படுகிறோம் O
     2. ஒத்திராதே O
     3. ஐயத்திற்கிடமான O
     4. ஒப்புக்கொள் O
     5. வலுவாக ஒப்புக்கொள்கிறேன் O
  2. நீங்கள் மீண்டும் அதே காரியத்தைச் செய்வீர்களா?
     1. முரண்படுகிறோம் O
     2. ஒத்திராதே O
     3. ஐயத்திற்கிடமான O
     4. ஒப்புக்கொள் O
     5. வலுவாக ஒப்புக்கொள்கிறேன் O

# Chapter 6 - Reference

1. Ministry of Health and indigenous medicine. National guideline for maternal care: Volume 1: 2013
2. Dutta DC. Text Book of Obstetrics. 6^th^ edition. New Central Book Agency. 2001. [ISBN](https://en.wikipedia.org/wiki/International_Standard_Book_Number) [978-81-7381-142-5](https://en.wikipedia.org/wiki/Special:BookSources/978-81-7381-142-5)
3. World Health Organization. Managing complication in pregnancy and childbirth: a guide for midwives and doctors. Geneva: World Health Organization; 2000. (available at: [http://www​.who.int/reproductivehealth​/publications​/maternal_perinatal_health​/9241545879/en/index​.html](http://www.who.int/reproductivehealth/publications/maternal_perinatal_health/9241545879/en/index.html))
4. Leduc D, Biringer A, Lee L, Dy J. Induction of labour. Journal of Obstetrics and Gynecology Canada. 2013; 35 (9): 840-857.
5. World Health Organization. WHO Handbook for Guideline Development. Geneva: World Health Organization; 2008.
6. National Institute of Clinical Excellence. Inducing labour, National Institute of Clinical Excellence; 2008. (available at: [http://www​.nice.org.uk/guidance/CG70](http://www.nice.org.uk/guidance/CG70)).
7. Boulvain M, Stan CM, Irion O. Membrane sweeping for induction of labour. Cochrane Database of Systematic Reviews 2005; 1(CD000451).

DOI: 10.1002/14651858.CD000451.pub2

1. Sri Lanka College of Obstetricians and Gynecologists. Guidelines of induction of labour. Sri Lanka Journal of Obstetrics and Gynecology. 2013. December: 136-138
2. Edmonds DK. Dewhurst’s Textbook of Obstetrics and Gynecology. 8^th^ edition. Wiley Blackwell. 2012. ISBN-13 978-0-470-65457-6
3. Standring S. Gray’s Anatomy: The anatomical basis of clinical practice. 41^st^ Edition. Elsevier. 2016. ISBN: 978-0-7020-5230-9
4. Lydon-Rochelle M, Holt VL, Easterling TR, Martin DP. Risk of uterine rupture during labor among women with a prior cesarean delivery. New England Journal of Medicine. 2001; 345 (1): 3-8.
5. Jozwiak M, Oude Rengerink K, Benthem M, van Beek E, Dijksterhuis MG, de Graaf IM, et al. Foley catheter versus vaginal prostaglandin E2 gel for induction of labour at term (PROBAAT trial): an open-label, randomised controlled trial. Lancet. 2011; 378 (9809): 2095-103.
6. Kehl S, Ehard A, Berlit S, Spaich S, Sutterlin M, Siemer J. Combination of misoprostol and mechanical dilation for induction of labour: a randomized controlled trial. European Journal of Obstetrics, Gynecology, and Reproductive Biology. 2011; 159 (2): 315-9.
7. Mackeen AD, Walker L, Ruhstaller K, Schuster M, Sciscione A. Foley catheter vs prostaglandin as ripening agent in pregnant women with premature rupture of membranes. The Journal of the American Osteopathic Association. 2014; 114 (9): 686-92.
8. Caughey AB, et al. Maternal and neonatal outcomes of elective induction of labor. Rockville, MD: Agency for Healthcare Research and Quality; 2009. Evidence Report/Technology Assessment No. 176. (Prepared by the Stanford University-UCSF Evidenced-based Practice Center) (AHRQ Publication No. 09-E005)
9. M D Mitchell, A P Flint, J Bibby, J Brunt, J M Arnold, A B Anderson, A C Turnbull. Rapid increases in plasma prostaglandin concentrations after vaginal examination and amniotomy. British Journal of Medicine. 1977; 2: 1183
10. [Gokhan Y](https://www.tandfonline.com/author/Yildirim%2C+Gokhan), [Kemal G](https://www.tandfonline.com/author/G%C3%BCng%C3%B6rd%C3%BCk%2C+Kemal), [Özge IK](https://www.tandfonline.com/author/Karada%C4%9F%2C+%C3%96zge+%C4%B0dem), [Halİl A](https://www.tandfonline.com/author/Aslan%2C+Hal%C4%B0l), [Erdem T](https://www.tandfonline.com/author/Turhan%2C+Erdem), [Yavuz C](https://www.tandfonline.com/author/Ceylan%2C+Yavuz). Membrane sweeping to induce labor in low-risk patients at term pregnancy: A randomised controlled trial. Journal of Maternal-Fetal and Neonatal Medicine. 2010: 23 (7): 681-687
11. [Helen A. Allott](https://obgyn.onlinelibrary.wiley.com/action/doSearch?ContribAuthorStored=Allott%2C+Helen+A), [Christopher R. Palmer](https://obgyn.onlinelibrary.wiley.com/action/doSearch?ContribAuthorStored=Palmer%2C+Christopher+R). Sweeping the membranes: a valid procedure in stimulating the onset of labour?. British Journal of Obstetrics and Gynecology. 1993: 100 (10): 898-903
12. Tan PC, Jacob R, Omar SZ. Membrane Sweeping at Initiation of Formal Labor Induction: A Randomized Controlled Trial. Obstetrics & Gynecology. [2006: 107 (3): 569-577](https://journals.lww.com/greenjournal/toc/2006/03000)
13. [Idrisa](https://www.tandfonline.com/author/Idrisa%2C+A) A, [Kyari](https://www.tandfonline.com/author/Kyari%2C+O) O, [Kawuwa](https://www.tandfonline.com/author/Kawuwa%2C+M+B) MB, [Usman](https://www.tandfonline.com/author/Usman%2C+H+A) HA. Fetal membrane sweeping for stimulation of labour in prolonged pregnancy. A controlled study. [Journal of Obstetrics and Gynaecology. 1993: 13 ([4](https://www.tandfonline.com/toc/ijog20/13/4)): 235-237](https://www.tandfonline.com/toc/ijog20/current)
14. [Ogbonmwan](https://www.tandfonline.com/author/Ogbonmwan%2C+S+E+O) SEO, [Miller](https://www.tandfonline.com/author/Miller%2C+V) V, [Ogbonmwan](https://www.tandfonline.com/author/Ogbonmwan%2C+D+E) DE, [Akinsola](https://www.tandfonline.com/author/Akinsola%2C+A+A) AA. Review of vaginal birth after primary caesarean section without prostaglandin induction and or syntocinon augmentation in labour. [The Journal of Maternal-Fetal & Neonatal Medicine. 2010: 23 (4): 281-285](https://www.tandfonline.com/toc/ijmf20/current)
15. [Tan PC](https://onlinelibrary.wiley.com/action/doSearch?ContribAuthorStored=Tan%2C+Peng+Chiong), [Khine PP](https://onlinelibrary.wiley.com/action/doSearch?ContribAuthorStored=Khine%2C+Pwint+Phyu), [Sabdin NH](https://onlinelibrary.wiley.com/action/doSearch?ContribAuthorStored=Sabdin%2C+Nur+Halimanja), [Vallikkannu N](https://onlinelibrary.wiley.com/action/doSearch?ContribAuthorStored=Vallikkannu%2C+Narayanan), [Sulaiman S](https://onlinelibrary.wiley.com/action/doSearch?ContribAuthorStored=Sulaiman%2C+Sofiah). Journal of Ultrasound in Medicine. 2011: [30 (2](https://onlinelibrary.wiley.com/toc/15509613/2011/30/2)): 227-233
16. Keller J, Ojo L, Sheth S, Young H, janakiraman V. Membrane sweeping in GBS positive patients: A randomized controlled trial. Oral Concurrent Sessions 7, 12/02/2011, San Francisco.

1. [L.C Foong](https://www.sciencedirect.com/science/article/pii/S0029784400009959#!), [K.V anaja](https://www.sciencedirect.com/science/article/pii/S0029784400009959#!), [G.Tan](https://www.sciencedirect.com/science/article/pii/S0029784400009959#!), [S Chua](https://www.sciencedirect.com/science/article/pii/S0029784400009959#!). Membrane sweeping in conjunction with labor induction. [Obstetrics & Gynecology](https://www.sciencedirect.com/science/journal/00297844). 2000: [96 (4](https://www.sciencedirect.com/science/journal/00297844/96/4)): 539-542
2. Hill MJ, McWilliams GD, Garcia-Sur DNP, Chen B, Munroe MCN, Hoeldtke NJ, The Effect of Membrane Sweeping on Pre-labor Rupture of Membranes: A Randomized Controlled Trial. Obstetrics & Gynecology. [2008: 111 (6): 1313-1319](https://journals.lww.com/greenjournal/toc/2008/06000)
3. Hamdan M, Sidhu K, Sabir N, Omar SZ, Tan PC, Serial Membrane Sweeping at Term in Planned Vaginal Birth After Cesarean: A Randomized Controlled Trial. Obstetrics & Gynecology. [2009: 114 (4): 745-751](https://journals.lww.com/greenjournal/toc/2009/10000)
4. Shafik A, Abou-Seeda M, Hofny M. Membrane sweeping prior to Induction of Labor: A Randomized Controlled Trial. Life Science Journal. 2014: 11: (3): 184-190
5. [Everett FM](https://www.nature.com/articles/7200133#auth-1), [Suneet PC](https://www.nature.com/articles/7200133#auth-2), [Michael FM](https://www.nature.com/articles/7200133#auth-3), [David JB](https://www.nature.com/articles/7200133#auth-4), [Colleen ME](https://www.nature.com/articles/7200133#auth-5), [John CM](https://www.nature.com/articles/7200133#auth-6). Membrane Sweeping versus Dinoprostone Vaginal Insert in the Management of Pregnancies beyond 41 Weeks with an Unfavorable Cervix. Journal of Perinatology. 1999: 19 (1): 88-91.
6. Julious SA. Sample size of 12 per group rule of thumb for a pilot study. Pharmaceutical Statistics. 2005: 4: 287-291. doi:[10.1002/pst.185](https://doi.org/10.1002/pst.185)
7. Kane SP. Sample size calculator. Clinicalc LLC.

Available on: <https://clincalc.com/stats/samplesize.aspx>

1. Baum N. Comprehension is the Key - The challenge hospitals face is that many patients do not understand the fundamental information regarding their treatment plans. Patient safety and quality healthcare. 2003. Available on: <https://www.psqh.com/mayjun06/informed.html>
2. Bierer B, Winkler S, Bernstein H, Myerson J, Kim M. Data safety monitoring Guidance. Harvard Catalyst: The Harvard Clinical and Translational Science Centre. Available on:

<https://catalyst.harvard.edu/pdf/regulatory/DSMB-P_Guidance.pdf>

1. Sekhon M, Cartwright M, Francis JJ. Acceptability of healthcare interventions: an overview of reviews and development of a theoretical framework. BMC Health Serv Res. 2017;17(1):88. doi:10.1186/s12913-017-2031-8
2. Wu M, McIntosh J, Liu J. Current prevalence rate of latex allergy: Why it remains a problem?. J Occup Health. 2016;58(2):138–144. doi:10.1539/joh.15-0275-RA
3. da Silva RM. Syncope: epidemiology, etiology, and prognosis. Front Physiol. 2014;5:471. Published 2014 Dec 8. doi:10.3389/fphys.2014.00471
4. Boulvain M, Stan CM, Irion O. Membrane sweeping for inductionof labour.Cochrane Database of Systematic Reviews2005,Issue 1. Art. No.: CD000451. DOI: 10.1002/14651858.CD000451.pub2
