# Supplementary material for: Comparing membrane sweep and cervical massage in preventing the need for formal labour induction in uncomplicated pregnancy at term: Secondary analysis of a randomized controlled trial
Source: PLoS One. 2025 May 20;20(5):e0324172. doi: 10.1371/journal.pone.0324172 (PMC12091732; doi:10.1371/journal.pone.0324172)
Supplement: Attachment — Submitted filename: Reviewers comments V2.docx [file pone.0324172.s006.docx]

02/07/2024

Editor

PLOS one,

Dear sir/ Madam

**Authors’ replies to the reviewer comments and resubmission of revised version of**

**PONE-D-24-07781**

We wish to thank the editors and the reviewers for their encouragement and helpful comments and suggestions for improvement on our paper titled “Comparing membrane sweep and cervical massage in preventing the need for formal labour induction in uncomplicated pregnancy at term: Secondary analysis of a randomized controlled trial”.

We have accepted and addressed all comments. The following is a point-by-point correction and edition list as per reviewer suggestions.

1. Reviewer 1 – “*briefly mention statistical methods in abstract*.” – Thank you. This has now been addressed in the manuscript- Line 30 - 42
2. Reviewer 1 – “*Define NNT”* – Defined. Line 32
3. Reviewer 2 – “*draw some parallels to balloon induction methods”* – Thank you. Discussion added - Line 111-115
4. Reviewer 2 – “*Request details about objective assessment of acceptability*”– Details added Line 170-188
5. Reviewer 1 – “*Include a detailed description of statistical methods employed”* – We have inserted a discussion on the statistical methods employed; Line 189-203.
6. Reviewer 1 – Table 6, 7 – “*Name the type of summary data presented”*, - thanks you, Column headings edited as such; Table 6,7.
7. Reviewer 1 – Table 9, 10 – “*Provide actual p value and not P>0.05, Give ratios and percentages”.* Done; Table 9,10.
8. Reviewer 2 – “*Comment about why we admitted the mothers for the procedures”* – Addressed in Line 307-310

Please be kind enough to review our resubmission for publication.

Thank you

Warm regards,

Madugalle TMSSB
