# Supplementary material for: Comparing membrane sweep and cervical massage in preventing the need for formal labour induction in uncomplicated pregnancy at term: Secondary analysis of a randomized controlled trial
Source: PLoS One. 2025 May 20;20(5):e0324172. doi: 10.1371/journal.pone.0324172 (PMC12091732; doi:10.1371/journal.pone.0324172)
Supplement: Attachment — Submitted filename: reponse to reviewers.docx [file pone.0324172.s007.docx]

Editors,

PLOS One,

Dear sir/madam,

**Author’s response for editorial and reviewer comments**

Thank you for your rapid response, encouragements and helpful comments on our submission numbered PONE-D-24-26866 and titled “Comparing membrane sweep and cervical massage in preventing the need for formal labour induction in uncomplicated pregnancy at term: Secondary analysis of a randomized controlled trial”.

We have accepted and addressed all the points raised by the editorial team and the esteemed reviewers.

Amendments made to fulfill journal requirements,

1. **Comment –**

Please ensure that your manuscript meets PLOS ONE's style requirements, including those for file naming. The PLOS ONE style templates can be found at
[https://journals.plos.org/plosone/s/file?id=wjVg/PLOSOne_formatting_sample_main_body.pdf](https://track.editorialmanager.com/CL0/https:%2F%2Fjournals.plos.org%2Fplosone%2Fs%2Ffile%3Fid=wjVg%2FPLOSOne_formatting_sample_main_body.pdf/1/010f019450e46f45-607caad6-91e6-4da9-9212-42c0399635c9-000000/HNpCve0SUR43tsvFKtM53DdvrT1hboB4wnasWcgZnoM=193) and
[https://journals.plos.org/plosone/s/file?id=ba62/PLOSOne_formatting_sample_title_authors_affiliations.pdf](https://track.editorialmanager.com/CL0/https:%2F%2Fjournals.plos.org%2Fplosone%2Fs%2Ffile%3Fid=ba62%2FPLOSOne_formatting_sample_title_authors_affiliations.pdf/1/010f019450e46f45-607caad6-91e6-4da9-9212-42c0399635c9-000000/g4tC72ifFEkRugcSr9K7zyACFEHdsUrrGgG94GT39-E=193)

**Response –**

Thank you. We have edited the manuscript to comply with the journal style requirements stipulated in the given guidelines.

2. **Comment –**

We note that the original protocol file you uploaded contains a confidentiality notice indicating that the protocol may not be shared publicly or be published. Please note, however, that the PLOS Editorial Policy requires that the original protocol be published alongside your manuscript in the event of acceptance. Please note that should your paper be accepted, all content including the protocol will be published under the Creative Commons Attribution (CC BY) 4.0 license, which means that it will be freely available online, and any third party is permitted to access, download, copy, distribute, and use these materials in any way, even commercially, with proper attribution.

Therefore, we ask that you please seek permission from the study sponsor or body imposing the restriction on sharing this document to publish this protocol under CC BY 4.0 if your work is accepted. We kindly ask that you upload a formal statement signed by an institutional representative clarifying whether you will be able to comply with this policy. Additionally, please upload a clean copy of the protocol with the confidentiality notice (and any copyrighted institutional logos or signatures) removed.

**Response –**

Thank you. This study was conducted fulfill the research component of the principal author’s training program leading to specialist qualification and board certification as a consultant obstetrician and gynaecologist. The Board of Study of Obstetrics and Gynaecology, Postgraduate Institute of Medicine (PGIM), University of Colombo Sri Lanka is a higher educational institution and it does not impose restriction on the studies conducted to fulfill the research requirement of the program. The protocol was assessed by two independent examiners appointed by the PGIM. Ethical clearance was granted by the Ethics Review Committee of the Faculty of Medicine, University of Colombo, Sri Lanka. There is no institutional restriction for making the anonymized study data and protocol to be published. The study data and the protocol are solely owned by the authors, and it was a self-funded study. The initial decision made by the authors was to make the anonymised data and the study protocol available upon reasonable request. We are happy to make the same available for public access under CC BY 4.0 in a data repository, and are now available in FigShare which is one of the recommended repositories suggested by PLOS One.

- Madugalle, Sameera; Jayawardane, Indu Asanka; Jayasundara, Chandana (2025). Data set multi.xlsx. figshare. Dataset. <https://doi.org/10.6084/m9.figshare.28243112.v1>
- Madugalle, Sameera; Jayawardane, Indu Asanka; Jayasundara, Chandana (2025). Data set primi.xlsx. figshare. Dataset. <https://doi.org/10.6084/m9.figshare.28243115.v1>
- B, Madugalle T. M. S. S.; S, Jayasundara D. M. C.; A, Jayawardane I. (2025). Questionnaire data excel version. figshare. Dataset. <https://doi.org/10.6084/m9.figshare.28248005.v1>
- S, Jayasundara D. M. C.; B, Madugalle T. M. S. S.; A, Jayawardane I. (2025). Study protocol - Comparing Effectiveness of and maternal Acceptability with uSing cErvicaL massage vs mEmbrane Sweep for cervical ripening in pregnant women at 38th week of geStation at a tertiary care unit. figshare. Online resource. <https://doi.org/10.6084/m9.figshare.28310540.v1>

We have uploaded a letter signed by the principal author clarifying the author’s decision to make the anonymised study data and study protocol public.

We have also removed the confidentiality notice pertaining to the publication of the study protocol and anonymized data and uploaded a clean copy.

3. **Comment**

We are unable to open your Supporting Information file [Data set primi.sav and Data set multi.sav]. Please kindly revise as necessary and re-upload.

**Response**

The .sav files contain the anonymised data of the study. These are save files that can be opened by IBM SPSS statistical software. We also have extracted the data onto excel files and uploaded together with the .sav files. In the response to comment Number 2, we have given the citations for the data sets. The excel files and the original SPSS .sav files have been uploaded as a part of resubmission.

4. **Comment**

We note that your Data Availability Statement is currently as follows: [All relevant data are within the manuscript and its Supporting Information files.]

**Response**

We have uploaded the study protocol and anonymised data sets as supporting files and have made them available in Figshare. We have provided links in the response to comment number 2.

We confirm at this time that we have submitted all the raw data required for replication of the study.

5. **Comment**

Please amend your authorship list in your manuscript file to include authors Dr. Thennakoon Mudiyanselage Salila Sameera Bandara Madugalle.

**Response**

Thank you. We have included Dr. Thennakoon Mudiyanselage Salila Sameera Bandara Madugalle’s name as surname with initials and he is the Principal and corresponding author.

6. **Comment**

Your ethics statement should only appear in the Methods section of your manuscript. If your ethics statement is written in any section besides the Methods, please move it to the Methods section and delete it from any other section. Please ensure that your ethics statement is included in your manuscript, as the ethics statement entered into the online submission form will not be published alongside your manuscript.

**Response**

Thank you. We have moved the ethical statement from the end of the manuscript to the end of the methods section.

7. **Comment**

Please include captions for your Supporting Information files at the end of your manuscript, and update any in-text citations to match accordingly.

**Response**

Thank you. We have renamed the supporting information files according to the provided guidance. We have changed in-text citations accordingly.

8. **Comment**

Please review your reference list to ensure that it is complete and correct. If you have cited papers that have been retracted, please include the rationale for doing so in the manuscript text, or remove these references and replace them with relevant current references. Any changes to the reference list should be mentioned in the rebuttal letter that accompanies your revised manuscript. If you need to cite a retracted article, indicate the article’s retracted status in the References list and also include a citation and full reference for the retraction notice.

**Response**

Thank you. We have rechecked the references list and confirmed that it is complete and correct. There are no retracted papers citied.

Amendments were actioned in response to reviewer comments.

**Reviewer 1**

1. **Comment**

In the Method section, please provide descriptions of statistical methods used for comparing outcome rates between groups.

**Response**

Thank you. We have added descriptions of statistical methods used to compare all the outcome rates between groups in the methods section.

2. **Comment**

Please correct “long-Rank mantel-Cox test” as “log-rank test”. Please note that it’s log-rank instead of long-rank. Since “log-rank test” is sometimes called “Mantel-Cox test”, you don’t have to put both names together.

**Response**

Thank you. We have made the corrections.

3. **Comment**

What’ RR and OR? How were they calculated? Please define acronyms the first time they appear in the text.

**Response**

Thank you. RR stands for relative risk and OR stands for odds ratio. Methods section contains a brief description of using contingency tables to calculate odds ratio and relative risk. We have defined acronyms at their first use with apologies in the resubmission – Line 194-195

4. **Comment**

In the results, it is better to use “RR=” and “95% CI=” rather than “RR-” or “95% CI-”.

**Response**

Thank you. We have made the suggested changes.

5. **Comment**

“due to small subgroup size” is not the reason for insignificant difference since multiparous group has even smaller size but with significant difference.

**Response**

Thank you. We have rephrased it.

6. **Comment**

In table 10, please add p values for each outcome. Those “0” p-values for comparing 0% vs. 0% are not correct. They should be close to 1.

**Response**

Thank you. We have included the missing probability values with apologies.

**Reviewer 2**

1. **Comment**

Abstract is longer than recommended 300 words and would benefit from having headings

**Response**

Thank you. We have rephrased and edited the abstract to conform with the journal requirements.

2. **Comment**

Some grammar amendments to improve readability

**Response**

Thank you. We have tweaked the text to make it more readable and grammatically accurate.

Thank you.

T. M. S. S. B. Madugalle

Principal and corresponding author
