# Supplementary material for: Comparing membrane sweep and cervical massage in preventing the need for formal labour induction in uncomplicated pregnancy at term: Secondary analysis of a randomized controlled trial
Source: PLoS One. 2025 May 20;20(5):e0324172. doi: 10.1371/journal.pone.0324172 (PMC12091732; doi:10.1371/journal.pone.0324172)
Supplement: Attachment — Submitted filename: Rebuttal letter.docx [file pone.0324172.s008.docx]

12/03/2025

Editors,

PLOS One,

Dear sir/madam,

**Rebuttal letter in response to editorial and reviewer comments - PONE-D-24-26866R1**

Thank you for your rapid response, encouragements and helpful comments on our submission numbered PONE-D-24-26866R1 and titled “Comparing membrane sweep and cervical massage in preventing the need for formal labour induction in uncomplicated pregnancy at term: Secondary analysis of a randomized controlled trial”.

We have accepted and addressed all the points raised by the editorial team and the esteemed reviewers.

- **Comment** - Please ask a biostatistician to help with professional writeup/edits in statistics methods and analysis results.

**Response** – Thank you. We have obtained the help of a biostatistician to make amendments/editions to the article.

- **Comment** - Lines 180-181, the sentence “…groups were compared using standard deviation, standard error of the mean, and t-tests to calculate probability (p) values at 95% confidence intervals.” Doesn’t described in a professional way. It may be revised as “…groups were compared using t-tests and P-values with 95% confidence intervals are reported.”

**Response** – Thank you. Amendments have been made in lines 189-194.

- **Comment** - The title of Table 7 should be revised as “P-values from Log rank tests for comparisons of “survival” curves after Membrane sweep (MS) versus Cervical massage.”

**Response** – Thank you. Edited.

- **Comment -** Through the text, since the studied end point is time to spontaneous labor rather than survival, to avoid confusion, I would replace the term “survival” without spontaneous labor using “Proportion of subjects without spontaneous labor”.

**Response** – Thank you. Editions made in table 4, Lines 200, 242, Fig 2 and 3 titles

- **Comment** - In Table 8, “P value at 95% CI” should be corrected as “P value”.

Response – Thank you. Edited.

- **Comment -** Lines 224-225, “Subgroup analysis according to parity did not show any statistically significant difference between any methods due to the small individual sub-group size.” I have mentioned last time, the reason of insignificance might not be due to small sample size. To be informative, please add more contents to Table 6 with the data in subgroups of parity to show if there are any potential differences in outcomes by parity.

Response – Thank you. Editions have been made. Additional data for Primi and multi subgroups have been included in Table 6.

- **Comment -** Lines 258-260, the conclusion based on overall data may be misleading. To give complete information, please add sentences to state that the shorter period only be significant in multiparous group.

**Response** – Thank you. Corrections made in line 321.

Thank you,

T. M. S. S. B. Madugalle

Principal and corresponding author
